# Interleukin‐37 improves T‐cell‐mediated immunity and chimeric antigen receptor T‐cell therapy in aged backgrounds

**DOI:** 10.1111/acel.13309

**Published:** 2021-01-22

**Authors:** Jamie A. G. Hamilton, Miyoung Y. Lee, Rae Hunter, Raira S. Ank, Jamie Y. Story, Ganesh Talekar, Talia Sisroe, Dov B. Ballak, Andrew Fedanov, Christopher C. Porter, Elan Z. Eisenmesser, Charles A. Dinarello, Sunil S. Raikar, James DeGregori, Curtis J. Henry

**Affiliations:** ^1^ Department of Pediatrics Emory University School of Medicine Atlanta GA USA; ^2^ Aflac Cancer and Blood Disorders Center Children’s Healthcare of Atlanta Atlanta GA USA; ^3^ Molecular and Systems Pharmacology Graduate Program Graduate Division of Biological and Biomedical Sciences Laney Graduate School Emory University School of Medicine Atlanta GA USA; ^4^ Emory University Atlanta GA USA; ^5^ Department of Biochemistry and Molecular Genetics University of Colorado Anschutz Medical Campus Aurora CO USA; ^6^ Department of Medicine Radboud University Medical Center Nijmegen The Netherlands; ^7^ Department of Medicine University of Colorado Anschutz Medical Campus Aurora CO USA; ^8^ Department of Immunology and Microbiology University of Colorado Anschutz Medical Campus Aurora CO USA; ^9^ Department of Pediatrics University of Colorado Anschutz Medical Campus Aurora CO USA

**Keywords:** aging, CAR T‐cells, cytokines, inflammation, leukemia, PD‐1, signaling, T‐cells

## Abstract

Aging‐associated declines in innate and adaptive immune responses are well documented and pose a risk for the growing aging population, which is predicted to comprise greater than 40 percent of the world's population by 2050. Efforts have been made to improve immunity in aged populations; however, safe and effective protocols to accomplish this goal have not been universally established. Aging‐associated chronic inflammation is postulated to compromise immunity in aged mice and humans. Interleukin‐37 (IL‐37) is a potent anti‐inflammatory cytokine, and we present data demonstrating that IL‐37 gene expression levels in human monocytes significantly decline with age. Furthermore, we demonstrate that transgenic expression of interleukin‐37 (IL‐37) in aged mice reduces or prevents aging‐associated chronic inflammation, splenomegaly, and accumulation of myeloid cells (macrophages and dendritic cells) in the bone marrow and spleen. Additionally, we show that IL‐37 expression decreases the surface expression of programmed cell death protein 1 (PD‐1) and augments cytokine production from aged T‐cells. Improved T‐cell function coincided with a youthful restoration of *Pdcd1*, *Lat*, and *Stat4* gene expression levels in CD4^+^ T‐cells and *Lat* in CD8^+^ T‐cells when aged mice were treated with recombinant IL‐37 (rIL‐37) but not control immunoglobin (Control Ig). Importantly, IL‐37‐mediated rejuvenation of aged endogenous T‐cells was also observed in aged chimeric antigen receptor (CAR) T‐cells, where improved function significantly extended the survival of mice transplanted with leukemia cells. Collectively, these data demonstrate the potency of IL‐37 in boosting the function of aged T‐cells and highlight its therapeutic potential to overcome aging‐associated immunosenescence.

## Graphical abstract

Aging is associated with chronic inflammation (inflammaging) and immunosenescence, which are thought to promote cancer development. Here, we demonstrate that treating aged mice with the anti‐inflammatory cytokine interleukin‐37 (IL‐37) abrogates inflammaging, suppresses the expression of immunoinhibitory proteins on aged T‐cells, and rejuvenates effector responses. Importantly, we found that recombinant IL‐37 treatment augments the efficacy of aged CAR T‐cells in murine models of leukemia.

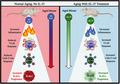

## 1 INTRODUCTION

Declining immunity is a hallmark of aging in mice and humans (Dorshkind et al., 2009; Henry et al., 2011). The effect of a waning immune response with age is thought to contribute to increased infection‐related mortalities in the elderly, higher cancer incidence, and decreased vaccination efficacy, which all pose major obstacles for maintaining a healthy aged population (Dorshkind et al., 2009; Henry et al., 2011).

The causes underlying aging‐associated immune impairments are under investigation with chronic inflammation being postulated as a major culprit responsible for compromising immunity and promoting aging‐associated diseases (Ahmad et al., 2009; Franceschi & Campisi, 2014; Licastro et al., 2005; Lin & Karin, 2007). “Inflammaging” in mice and humans is characterized by a subclinical, systemic increase in pro‐inflammatory cytokines including tumor necrosis factor‐alpha (TNF‐α), interleukin‐6 (IL‐6), interleukin‐1 beta (IL‐1β), and C‐reactive protein (CRP) (Franceschi & Campisi, 2014; Frasca & Blomberg, 2016; Mejias et al., 2018; Olivieri et al., 2018). Elevated levels of these inflammatory mediators have been shown to regulate the homeostasis and function of hematopoietic stem, progenitor, and mature immune cells, which express cytokine receptors that regulate their steady and activated states (de Bruin et al., 2013; Passegue & Ernst, 2009; Pronk et al., 2011; Qin et al., 2019; Sato et al., 2009; Schuettpelz & Link, 2013; Yamashita & Passegue, 2019).

The impact of aging‐associated immune senescence and chronic inflammation on the safety and efficacy of immune‐based therapies has not been thoroughly investigated. Immunotherapies have revolutionized our ability to treat refractory and relapsed diseases (Bayraktar et al., 2019; Boettcher et al., 2019; Riker et al., 2007; Zhang & Chen, 2018). Antibody‐mediated and chimeric antigen receptor (CAR) T‐cell therapies have shown remarkable success in treating previously intractable diseases such as melanoma (Riker et al., 2007). Immunotherapies are also frequently used to treat relapsed and refractory hematological malignancies including B‐cell acute lymphoblastic leukemia (B‐ALL) and diffuse large B‐cell lymphoma (DLBCL) (Barsan et al., 2020; Davila & Brentjens, 2016; Jacoby et al., 2019; Pehlivan et al., 2018). Despite the success of immunotherapies in patients with terminal disease, over 50% of patients receiving CD19‐directed CAR T‐cell therapy will relapse within the first 2 years of receiving treatment, which is attributed, in part, to the loss of the target antigen on malignant cells (Cao et al., 2018; Cheng et al., 2019; Gardner et al., 2017; Lee et al., 2015; Li, Zhang, et al., 2018; Maude, Frey, et al., 2014; Maude et al., 2018; O'Donnell et al., 2019; Park et al., 2018; Song et al., 2019; Turtle et al., 2016). Other factors that may contribute to the efficacy of immunotherapies include the inflammatory status of the patient and inherent quality of the immune system, including T‐cells.

We have previously demonstrated that the anti‐inflammatory cytokine interleukin‐37 (IL‐37) reduces aging‐associated inflammation and improves hematopoiesis in aged mice (Henry et al., 2015). There are over a dozen interleukin‐1 (IL‐1) family members that mainly act to promote inflammation (e.g., IL‐1α, IL‐1β, and IL‐18). However, interleukin‐37 (IL‐37) is a relatively new family member capable of blocking the pro‐inflammatory actions of IL‐18 by competing for the IL‐18 receptor (IL‐18Rα subunit) and attenuating MyD88 activity when it binds to the Ig‐like Toll/IL‐1R (TIR) receptor known as TIR8 (Dinarello et al., 2016; Eisenmesser et al., 2019; Nold et al., 2010). There are five different IL‐37 splice variants encoded by humans (denoted IL‐37a‐e); however, IL‐37b is the predominant form found in humans (Dinarello & Bufler, 2013; Dinarello et al., 2016). Thus, IL‐37b is commonly referred to simply as IL‐37 (Dinarello & Bufler, 2013). Messenger RNA (mRNA) for IL‐37 has been found in various human tissues including the bone marrow, lung, thymus, and lymph nodes and is produced by activated dendritic cells (DCs), natural killer (NK) cells, monocytes, and B‐cells (Dinarello & Bufler, 2013). Studies performed in IL‐37 transgenic (IL‐37tg) mice reveal that IL‐37 potently suppresses IL‐6, IL‐1β, and TNF‐α production in response to various TLR agonists or diseases driven by chronic inflammation including atherosclerosis, hepatocellular carcinoma, and colitis (Ji et al., 2017; Liu et al., 2016; Liu et al., 2018; McNamee et al., 2011; Nold et al., 2010; Zhao et al., 2014).

Given the close association of aging, declining immunity, and cancer development, in this study we determined how IL‐37 impacted the function of aged endogenous and CAR T‐cells. We demonstrate that transgenic expression of IL‐37 in aged mice and treating aged mice with recombinant human IL‐37 (rIL‐37) improves the function of non‐engineered and CAR T‐cells. To the best our knowledge, our results are the first to demonstrate that treating aged mice with rIL‐37 restores the expression of key genes involved in T‐cell activation which decline with normal aging and reduces the surface expression of multiple immunoinhibitory proteins on aged CD4^+^ and CD8^+^ T‐cells to youthful levels. Furthermore, we demonstrate that IL‐37 signaling directly opposes TNF‐α signaling and downregulates PD‐1 surface expression on aged T‐cells. Additionally, rIL‐37 treatment of aged mice augments cytokine production by endogenous T‐cells, and when combined with CAR T‐cell therapy, improves their therapeutic capacity in a murine model of B‐ALL. Given our findings that the expression of the IL‐37 gene decreases in an age‐dependent manner in human monocytes, our results demonstrate that increasing circulating IL‐37 levels in aged backgrounds may represent a novel strategy to overcome aging‐associated T‐cell senescence.

## 2 RESULTS

### 2.1 Interleukin‐37 suppresses inflammaging, and decreased levels are observed in aged human monocytes

One hallmark of aging is the onset of chronic inflammation in mice and humans (Frasca & Blomberg, 2016). This manifestation is postulated to contribute to numerous aging‐associated pathologies including cancer (Ferrucci & Balducci, 2008; Frasca & Blomberg, 2016; Furman et al., 2019; Leonardi et al., 2018). The underlying mechanisms governing aging‐associated chronic inflammation are being investigated, with data supporting reduced gut barrier function and microbiome dysbiosis emerging as plausible explanations (Biragyn & Ferrucci, 2018; Fernandes et al., 2019; Fransen et al., 2017). Furthermore, studies performed on aged monocytes and macrophages demonstrated more robust and durable inflammatory responses when myeloid cells were stimulated (Biragyn & Ferrucci, 2018; Kovtonyuk et al., 2016; Puchta et al., 2016); however, the reasons behind these heightened responses are unclear.

**FIGURE 1:**
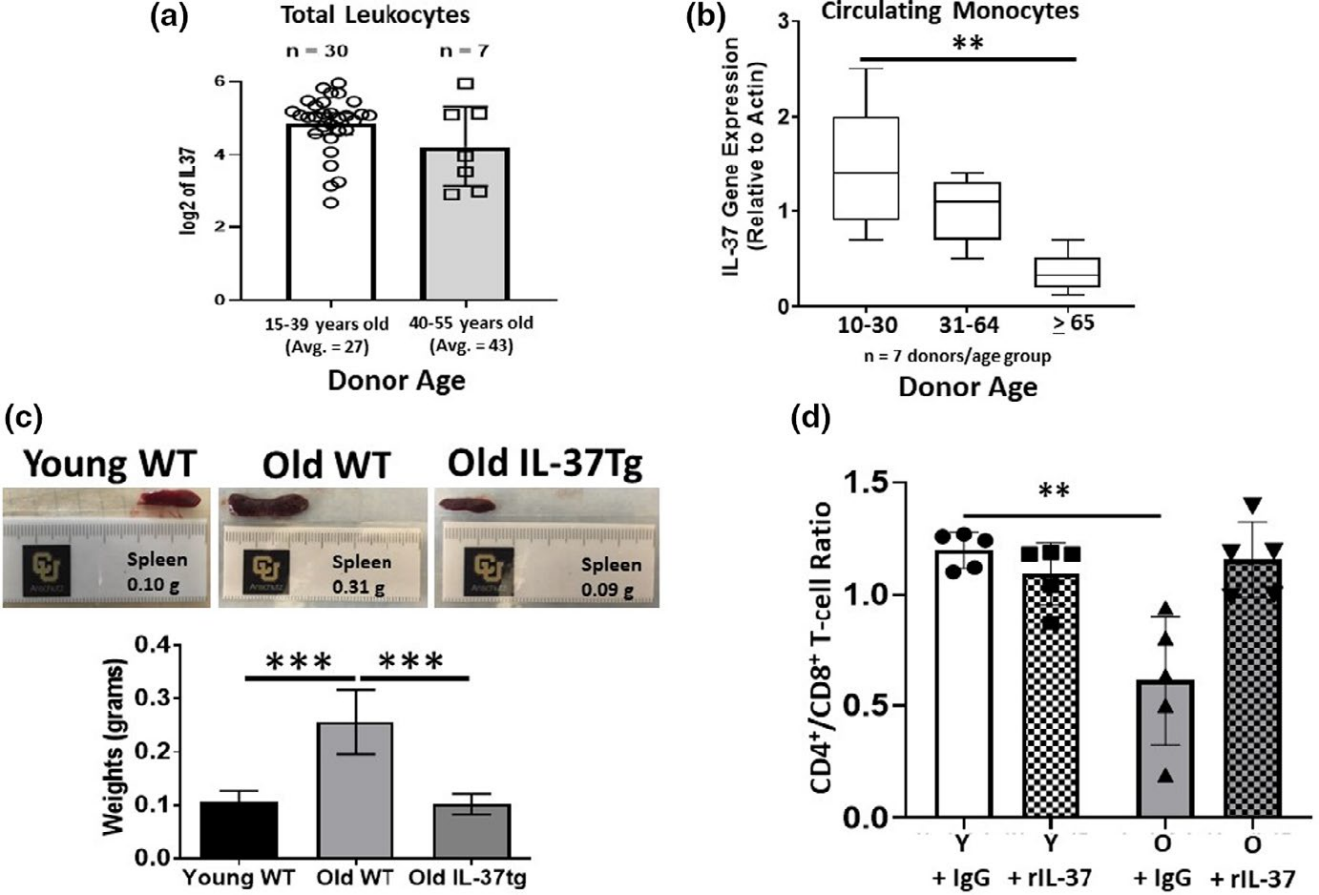
Interleukin‐37 suppresses inflammaging, and decreased levels are observed in aged human monocytes. (a) The R2 Database was mined to determine *IL‐37* gene expression levels in healthy donors between the ages of 1555 years of age. The gene expression levels are shown for young and middle‐aged donors. (b) Monocytes were purified from PBMCs of healthy donors using MACs selection. The gene expression levels are shown for young, middle‐aged, and old donors. (c) C57BL/6 wild‐type and IL‐37 transgenic mice were aged for 24 months and dissected to observe potential anatomical changes. The spleen appearance and weight are shown. (d) Young (2 months) and old (24 months) C57BL/6 mice were treated every other day for 2 weeks with control Ig or rIL‐37, and the ratio of CD4^+^ to CD8^+^ T‐cells was determined via flow cytometric analysis. Means ± *SD* are shown with **p < 0.01 and ***p < 0.001 determined using a Student's *t* test relative to young donors in *B* or young Control Ig‐treated mice in (d). A one‐way ANOVA with Tukey's post‐test was used to determine significance in (c). For results presented in (c), 3 independent experiments were conducted (*n* = 9 mice/group). In (d), data represent 5 mice/group

We have previously demonstrated that transgenic expression of the anti‐inflammatory cytokine IL‐37 improves hematopoiesis and the function of B‐progenitor cells in aged mice, which was largely driven by reducing aging‐associated inflammation (Henry et al., 2015). We next wanted to determine how treating aged mice (≥24 months old) with rIL‐37 impacted systemic inflammation relative to levels observed in IL‐37 transgenic (IL‐37 Tg) mice. Aged (24 months old) wild‐type mice were treated with control immunoglobulin (Control Ig) or rIL‐37 every 2 days for 2 weeks. We found that rIL‐37 treatment significantly decreased circulating tumor necrosis factor‐alpha (TNF‐α; Figure S1A), interleukin‐1 beta (IL1β; Figure S1B), and interleukin‐6 (IL‐6; Figure S1C) levels in aged mice which were comparable to observations in aged IL‐37tg mice (Figure S1D–F). Given the ability of IL‐37 to mitigate inflammaging in aged mice, we next asked whether IL‐37 levels declined in humans with age. We mined the R2 database for studies where IL‐37 gene expression profiles were available for healthy donors. Based on this criterion, we analyzed a repository submitted by Tompkins *et al*. The age range of donors in this database was 15–55 years old, reflective of young to middle‐aged healthy humans (Figure S2). We arbitrarily set the cutoff for young donors as those between 15 and 39 years of age and middle‐aged as donors between 40–55 years of age (Figure S2). When the data were binned into these groups, we found a slight decrease in IL‐37 gene expression levels in leukocytes recovered from middle‐aged (*n* = 7) relative to young donors (*n* = 30) (Figure 1a). To assess the impact of advanced age on IL37 expression levels, we obtained peripheral blood mononuclear cells (PBMCs) from healthy donors of various ages, including those over 65 years of age. Monocytes, which are major producers of IL‐37 (Cavalli & Dinarello, 2018; Li et al., 2019; Rudloff et al., 2017), were purified from PBMCs, and IL‐37 and actin gene expression levels were compared. Similar to data provided in the Tompkins *et al*. study, we found a decreased trend in IL‐37 gene expression levels in monocytes isolated from donors between 10 and 30 and those 31–64 years of age (Figure 1b). In donors 65 and older, IL‐37 gene expression levels in monocytes were significantly lower than those observed in monocytes isolated from donors between 10 and 30 years of age. In all, these data demonstrate the potent ability of IL‐37 to suppress aging‐associated chronic inflammation and suggest that reduced IL‐37 levels in aged monocytes may contribute to the onset of inflammaging in humans.

### 2.2 Interleukin‐37 abrogates splenomegaly and restores a youthful T‐cell distribution in aged mice

Given these observations and our previous studies demonstrating improved B‐progenitor cell function in IL‐37 transgenic (IL‐37 Tg) mice (Henry et al., 2015), we next determined whether recombinant IL‐37 (rIL‐37) treatment of aged mice mitigated aging‐associated changes in hematopoiesis. Aged (24 months old) wild‐type mice were treated with control immunoglobulin (Control Ig) or rIL‐37 every 2 days for two weeks (Figure S3A). We found that rIL‐37 treatment in aged mice prevented the aging‐associated accumulation of myeloid progenitor cells in the bone marrow (Figure S3D) and macrophages in the spleen (Figure S3E). Despite altering the relative distribution of myeloid cells, rIL‐37 treatment did not change the absolute number of hematopoietic stem cells (Figure S3B), B‐progenitor cells (Figure S3C), splenic‐derived B‐cells (Figure S3E), or splenic‐derived T‐cells (Figure S3E) in aged mice.

Aging is associated with extensive microarchitectural changes in the spleen including the onset of splenomegaly as a result of prolonged stimulation mediated by chronic inflammation or neoplastic lesions (Aw et al., 2016; Pettan‐Brewer & Treuting, 2011). In addition to abrogating aging‐associated chronic inflammation, we found that transgenic expression of IL‐37 also significantly reduced splenomegaly in aged mice (Figure 1c). Similar to its impact on hematopoiesis, treating aged mice with rIL‐37 also mitigated splenomegaly (data not shown). Given this observation, we next determined how rIL‐37 treatment of aged, naïve mice impacted the distribution of splenic‐derived immune cells and their basal activation state. Young (2 months old) and aged (24 months old) mice were treated with control immunoglobin (Control Ig) or rIL‐37 using the protocol described above. In these experiments, we found similar percentages of splenic‐derived CD4^+^ T‐cells in young, naïve mice treated with Control Ig and rIL‐37 (Figure S4A,B). In aged mice treated with Control Ig, we observed a slight decrease in the percentage of splenic‐derived CD4^+^ T‐cells relative to all treatment groups, whereas rIL‐37 treatment led to a noticeable, although not statistically significant (*p* = 0.059), increase in the representation of T‐helper cells (Figure S4A,B). Similarly, equivalent percentages of splenic‐derived CD8^+^ T‐cells were observed in young, naive mice treated with Control Ig and those treated with rIL‐37 (Figure S5A,B). In contrast to the slight decrease in the representation of CD4^+^ T‐cells observed in aged mice treated with Control Ig, the percentage of CD8^+^ T‐cells was noticeably, yet insignificantly (*p* = 0.084), higher than those observed in all treatment groups (Figure S5A,B). Interestingly, the trend toward increased representation of splenic‐derived CD8^+^ T‐cells in aged mice was mitigated by rIL‐37 treatment (Figure S5A,B). Overall, these data demonstrate that treating aged mice with rIL‐37 abrogates aging‐associated splenomegaly and restores the representation of CD4^+^ and CD8^+^ T‐cells to youthful levels (Figure 1d), whereas treating young mice with this anti‐inflammatory cytokine does not impact the distribution of splenic‐derived T‐cells.

### 2.3 IL‐37 promotes youthful gene expression profiles in aged T‐cells and reduces the surface expression of immunoinhibitory proteins

**FIGURE 2:**
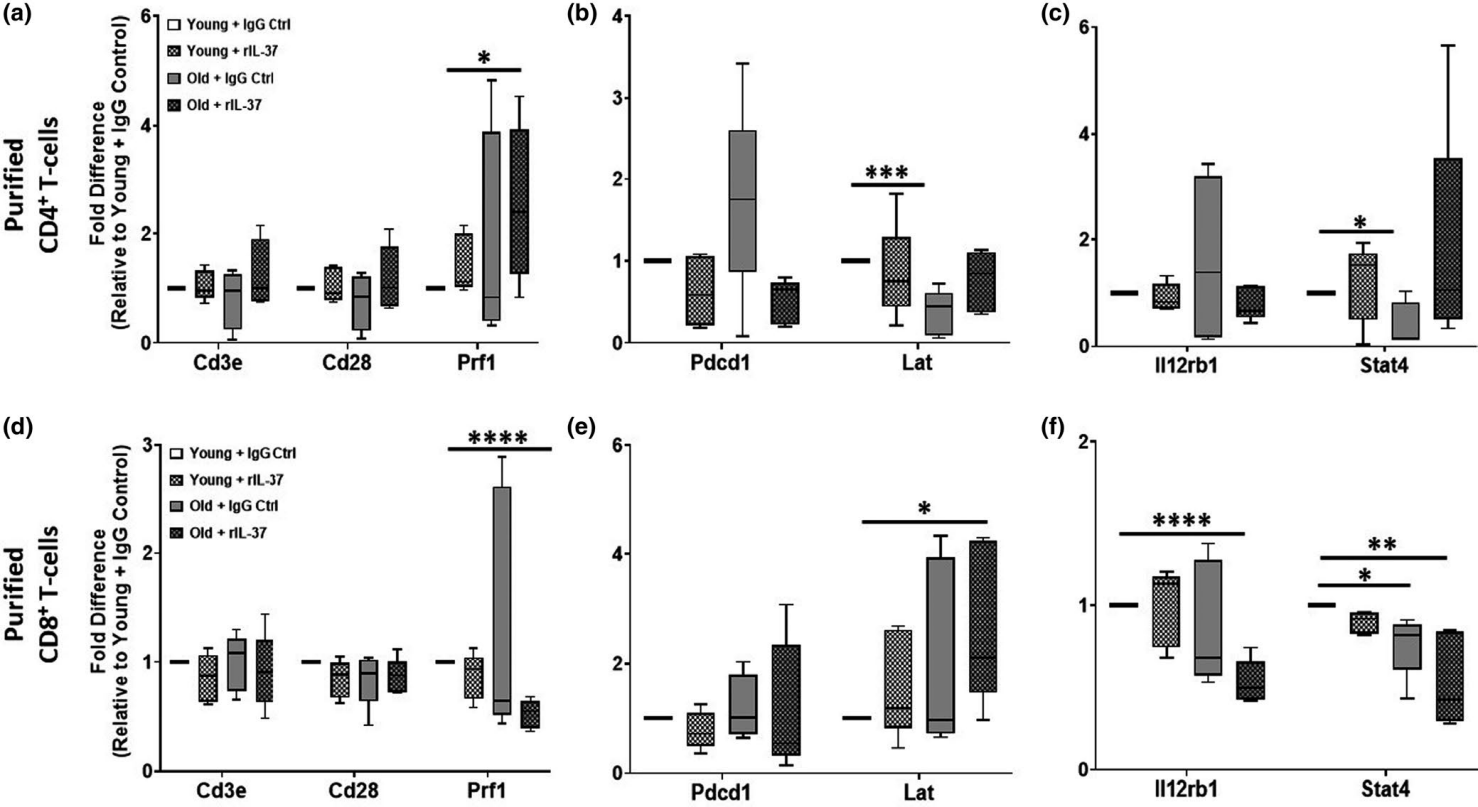
Interleukin‐37 restores a youthful gene expression profile in aged T‐cells. (a‐f) Young (2 months) and old (24 months) C57BL/6 mice (*n* = 5 mice/group) were treated every other day for 2 weeks with control Ig or rIL‐37. Naïve T‐cells were purified, RNA isolated, and qPCR analysis was performed to ascertain the steady‐state levels of genes involved in T‐cell activation (*Cd3e*, *Cd28*, *Prf1*, *Lat*, *Il12rb1*, *Stat4*) and inhibition (*Pdcd1*). Significance was determined using a one‐way ANOVA with Tukey's post‐test with *p < 0.05, **p < 0.01, ***p < 0.001, and ****p < 0.0001

Given the ability of rIL‐37 treatment to restore a youthful CD4^+^ to CD8^+^ T‐cell distribution in aged mice, we next determined how treatment with this anti‐inflammatory cytokine impacted the gene and surface expression of regulators of T‐cell activation. After 2 weeks of treatment, CD4^+^ T‐cells isolated from aged mice treated with rIL‐37 exhibited gene expression profiles that phenocopied CD4^+^ T‐cells isolated from young mice (Figure 2a–c). When aged mice were treated with Control Ig, CD4^+^ T‐cells exhibited a trend toward higher gene expression levels of *Pdcd1* (the gene encoding programmed cell death protein 1 [PD‐1]) and significantly lower levels of *Lat* and *Stat4* (Figure 2b,c). Treatment of aged mice with rIL‐37 reversed these phenotypes in CD4^+^ T‐cells to youthful levels, which was comparable to young mice treated with Control Ig and rIL‐37 (Figure 2b,c). Furthermore, we observed a significant increase in *Prf1* (the gene encoding perforin) expression levels in CD4^+^ T‐cells isolated from aged mice treated with rIL‐37. Unlike rIL‐37‐mediated gene expression alterations in aged CD4^+^ T‐cells, treating young mice with rIL‐37 did not alter *Cd3e*, *Cd28*, *Prf1*, *Pdcd1*, *Lat*, *Il12rb1*, or *Stat4* gene expression levels in CD4^+^ T‐cells (Figure 2a–c). In addition to altering gene expression profiles in aged CD4^+^ T‐cells, treating aged mice with rIL‐37 also decreased the surface expression of the immunoinhibitory proteins Tim‐3 and TIGIT on aged T‐cells, whereas CD28 surface levels remained unchanged (Figure S4C–F). Despite the aging‐associated increase in *Pdcd1* gene expression levels in aged CD4^+^ T‐cells (Figure 2b), PD‐1 surface expression on naïve CD4^+^ T‐cells was negligible and not impacted by rIL‐37 treatment (Figure S4C), which is consistent with PD‐1 expression being induced on activated T‐cells (Riley, 2009).

Unlike, changes observed in CD4^+^ T‐cells in aged mice receiving rIL‐37 treatment, aging‐associated gene expression changes in CD8^+^ T‐cells were largely unchanged with rIL‐37 treatment with the exception of restoring youthful levels of *Lat* (Figure 2d–f). Similarly to CD4^+^ T‐cells, treating aged mice with rIL‐37 also significantly decreased TIGIT surface levels on naïve CD8+ T‐cells (Figure S5C,E), whereas Tim3 (Figure S5C,D) and CD28 (Figure S5C,F) surface expression was not impacted by rIL‐37 treatment in young or aged mice.

In addition to assessing the impact of rIL‐37 treatment on aged T‐lymphocytes, we also determined its impact on aged myeloid cells. Treating aged mice with rIL‐37 also led to a reduction (albeit insignificant) in splenic dendritic cells (Figure S6A,B) and macrophages (Figure S6C,D), consistent with an abrogation of splenomegaly (Figure 1c and Figure S3D,E). Despite decreasing the percentages of splenic‐derived dendritic cells, which are the principal activators of naïve T‐cells (Henry et al., 2008, 2010), rIL‐37 treated did not rejuvenate their upregulation of the costimulatory molecules CD40, CD80, and CD86 to youthful levels after *ex vivo* stimulation with LPS (data not shown).

In all, these data demonstrate that treating aged mice with rIL‐37 alters the activation threshold of naïve CD4^+^ and CD8^+^ T‐cells, by increasing the expression of genes involved in T‐cell activation (*Stat4* and *Lat*) and decreasing the surface expression of immunoinhibitory proteins (Tim‐3 and TIGIT).

### 2.4 Recombinant IL‐37 treatment improves T‐cell function in aged mice

**FIGURE 3:**
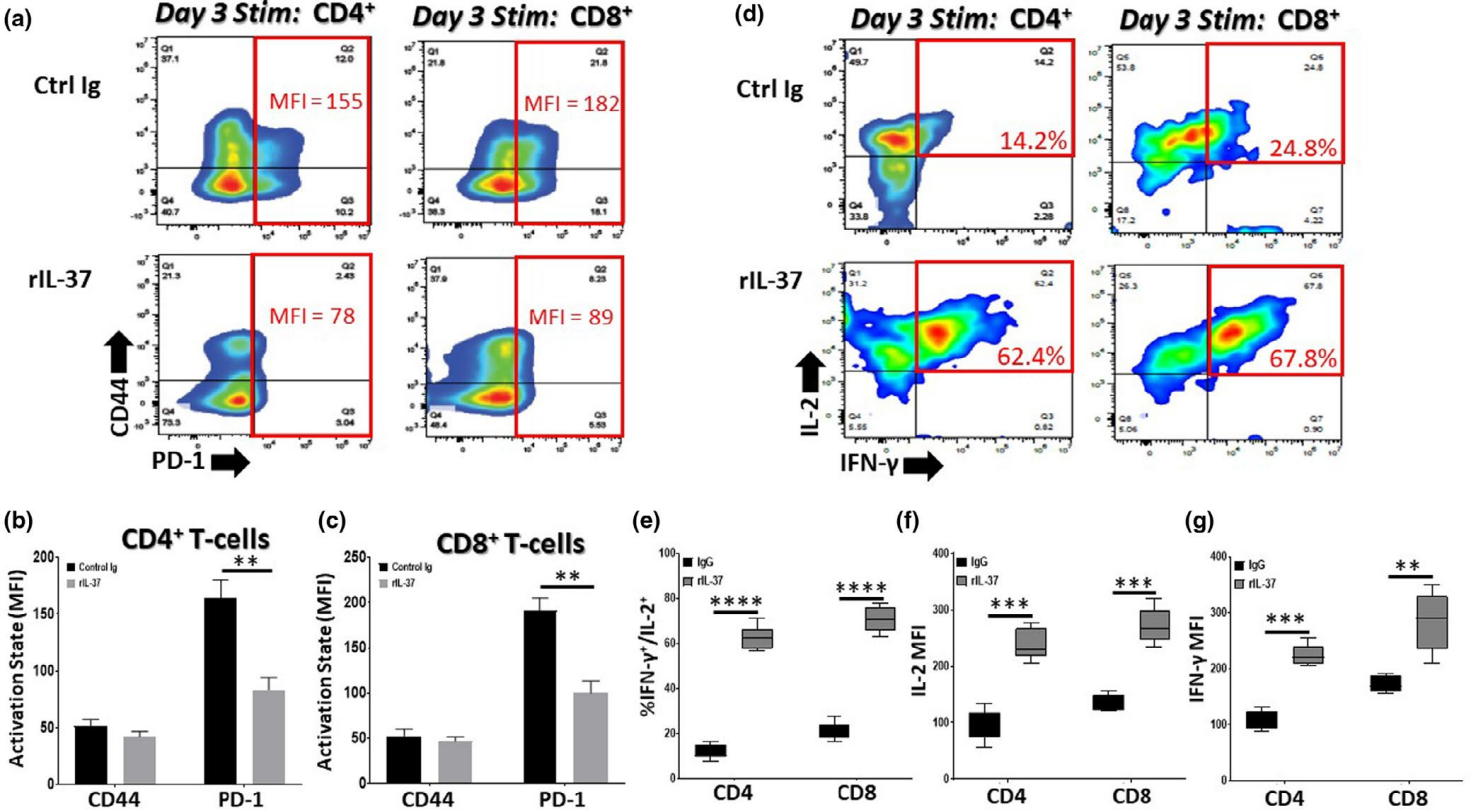
Recombinant IL‐37 treatment reduces PD‐1 surface expression and improves the function of aged T‐cells. Aged (24 months old) C57BL/6 mice were treated with control immunoglobulin (Control Ig) or recombinant IL‐37 (rIL‐37) every other day for 2 weeks. Naïve CD4^+^ T‐cells and CD8^+^ T‐cells were purified from treated mice using MACs selection and stimulated in vitro with αCD3/αCD28. On day 3 post‐stimulation, (a–c) the mean surface expression of CD44 and PD1 (mean fluorescence intensity [MFI]) and (d–g) the percentage and MFI of IL‐2/IFN‐γ‐producing T‐cells were determined using flow cytometric analysis. Means ± *SD* are shown in (b, c, e, f, and g) with **p < 0.01, ***p < 0.001, and ****p < 0.0001 determined using a Student's *t* test relative to aged T‐cell responses from Control Ig treated mice. *n* = 9 mice/group with 3 independent experiments conducted. The red boxes in (a) and (d) denote functional parameters of interest

Given that IL‐37 treatment rejuvenated gene expression profiles and suppressed the surface expression of immunoinhibitory proteins, we next assessed how T‐cell function was impacted. We purified CD4^+^ and CD8^+^ T‐cells from aged (24 months old) wild‐type mice treated every other day for 2 weeks with Control Ig or rIL‐37 and stimulated them in vitro with αCD3/αCD28 for 3 days. We found that rIL‐37 treatment significantly mitigated T‐cell exhaustion indicative of similar T‐cell expansion observed between T‐cell isolated from aged mice treated with rIL‐37 and young mice treated with Control Ig or rIL‐37 (Figure S7A,B). In contrast, T‐cell proliferative defects were observed in aged T‐cell isolated from aged mice treated with Control Ig, where significant difference were apparent by Day 2 of culture and became more pronounced by Day 4 post‐stimulation (Figure S7A,B). Furthermore, treating aged mice with rIL‐37 significantly reduced the surface expression of PD‐1 on effector CD4^+^ and CD8^+^ T‐cells, whereas CD44 surface levels remain unchanged (Figure 3a–c). We found that T‐cells stimulated from aged mice treated with rIL‐37 were more functional than T‐cells activated from aged mice treated with Control Ig (Figure 3d). We observed significant increases in interleukin‐2 (IL‐2) and interferon‐gamma (IFN‐γ) production at the population (percentage; Figure 3e) and per cell (mean fluorescence intensity; Figure 3f,g) levels when T‐cells were stimulated *ex vivo* from rIL‐37‐treated but not Control Ig‐treated aged mice. In summary, these data demonstrate that treating aged mice with recombinant IL‐37 effectively improves T‐cell responses.

**FIGURE 4:**
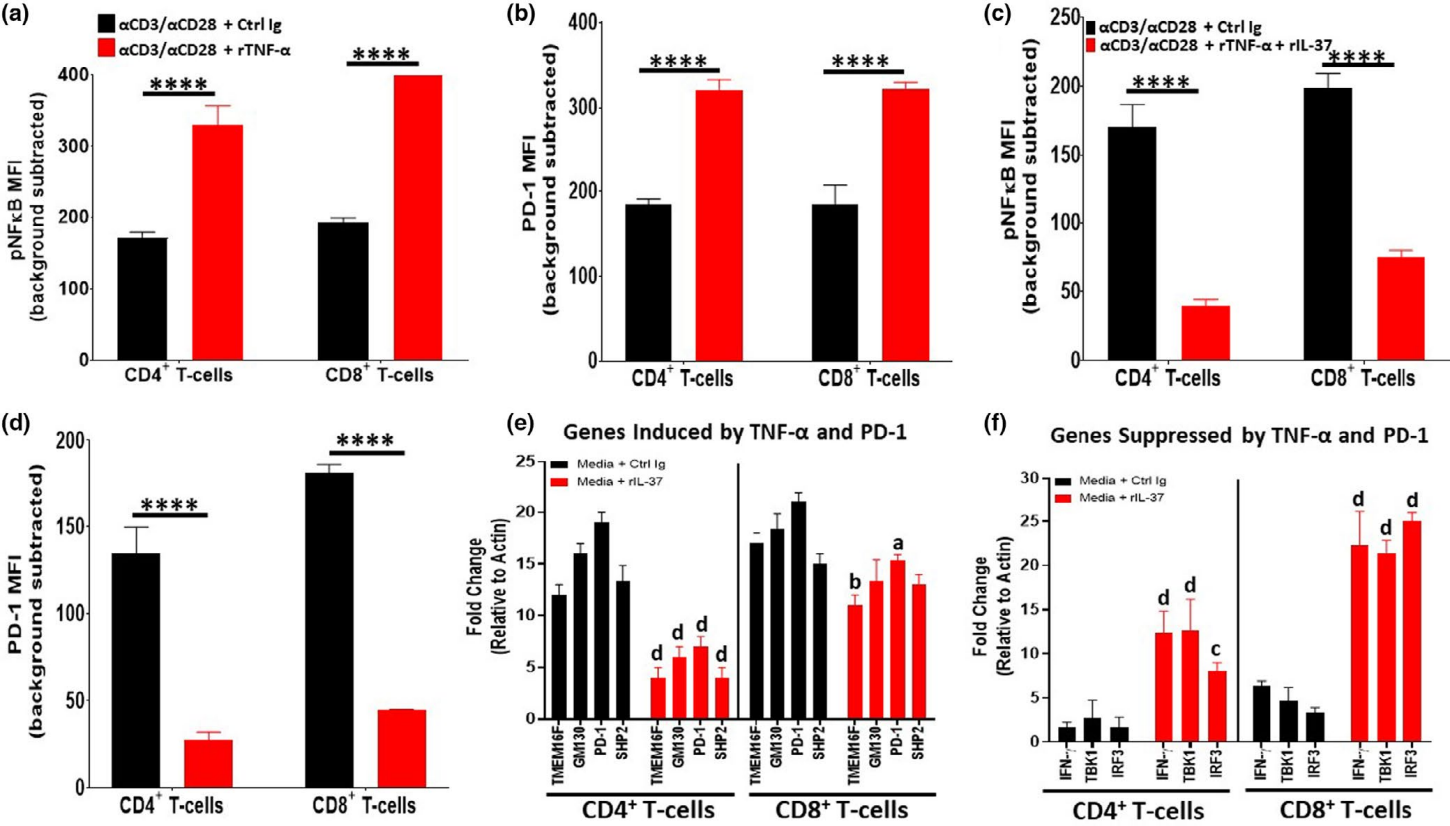
Recombinant IL‐37 treatment opposes TNF‐α signaling in aged T‐cells. Naïve CD4^+^ and CD8^+^ T‐cells were purified from aged (24 months old) C57BL/6 mice via MACs selection and stimulated in vitro with αCD3/αCD28 in the presence of Control Ig, rTNF‐α, or rTNF‐α + rIL‐37. (a, c) After 10’ of stimulation, phospho‐flow cytometry was performed to determine NF‐κB activation. (b, d) After 3 days of stimulation, the surface expression of PD‐1 on aged T‐cells was determined using flow cytometric analysis. (e, f) Naïve T‐cells were purified as described above and stimulated with Control Ig or rIL‐37 for 4 h. After the stimulation period, qPCR analysis was performed to ascertain the expression levels of genes involved in T‐cell activation (IFN‐γ, TBK1, IRF3) and inhibition (TMEM16F, GM130, SHP2, and PD1). Importantly, the genes chosen for assessment are regulated by TNF‐α and PD‐1 signaling. Significance was determined using a Student's *t* test relative to αCD3/αCD28 + Control Ig (a–d) and media +Control Ig (e, f) treated groups. For a–d, means ± *SD* are shown with ****p < 0.0001. For (e) and (f), **a**=*p < 0.05, **b**=**p < 0.01, **c**=***p < 0.001, and **d**=****p < 0.0001 where gene expression levels observed in Control Ig‐treated aged T‐cells were used as the positive control for each gene tested. *n* = 9 mice/group with 3 independent experiments conducted

Pro‐inflammatory cytokines, such as TNF‐α, are potent inducers of PD‐1/PD‐L1 surface expression on immune cells (Bally et al., 2015; Lu et al., 2019). Given that treating aged mice with rIL‐37 significantly reduced chronic inflammation and was particularly effective at lowering circulating TNF‐α levels (Figure S1A,D), we next determined if rIL‐37 directly counteracted TNF‐α signaling and its ability to induce PD‐1 surface expression on aged T‐cells. In immune cells, TNF‐α is a potent inducer of NF‐κB activation (Liu et al., 2017) and NF‐κB binding sites are located in the PD‐1 promoter (Redd et al., 2018). In these studies, we found that treating aged CD4^+^ and CD8^+^ T‐cells with recombinant TNFα (rTNF‐α) significantly augmented NF‐κB activation in T‐cells (Figure 4a and Figure S8A) which correlated with increased PD‐1 surface expression on effector T‐cells (Figure 4b). We next determined whether rIL‐37 stimulation could reduce NF‐κB activation in TNF‐α stimulated aged T‐cells. Interestingly, we found that rIL‐37 abrogated the TNF‐α induced NF‐κB activation in aged T‐cells (Figure 4c and Figure S8B) and significantly decreased PD‐1 surface expression (Figure 4d).

To determine whether IL‐37 altered T‐cell homeostasis prior to stimulation, we next performed gene expression profiling of targets induced (TMEM16F, GM130, PD‐1, and SHP2) and suppressed (IFN‐γ, TBK1, and IRF3) by TNF‐α and PD‐1 signaling in aged naïve T‐cells treated with Control Ig or rIL‐37. In young naïve T‐cells, we observed low basal expression of genes induces and suppressed by TNF‐α and PD‐1 signaling (Figure S8C). Furthermore, rIL‐37 treatment did not impact the expression of these genes in young naïve T‐cells (Figure S8D). In contrast, aged naïve T‐cells exhibited high gene expression levels of TMEM16F, GM130, PD1, and SHP2 suggesting that these programs are primed for induction in aged T‐cells (Figure 4e). Furthermore, treating aged naïve T‐cells with rIL‐37 significantly increased the homeostatic expression of genes suppressed by TNF‐α and PD‐1 signaling, particularly those involved in interferon production (Figure 4f). Taken together, these data demonstrate that rIL‐37 improves the function of aged T‐cells which is mediated, in part, by the ability of rIL37 treatment to directly oppose TNF‐α‐induced programs in aged T‐cells.

### 2.5 Recombinant IL‐37 treatment protects aged mice from B‐ALL pathogenesis in a T‐cell dependent manner

Two hallmarks of aging are the onset of chronic inflammation and compromised immunity, which are postulated to contribute to numerous aging‐associated pathologies including cancer (Ferrucci & Balducci, 2008; Furman et al., 2019; Leonardi et al., 2018). We have previously demonstrated that transgenic expression of IL‐37 improves hematopoiesis and the function of B‐progenitor cells in aged mice, which was largely driven by reducing aging‐associated inflammation (Henry et al., 2015). To determine how reducing aging‐associated chronic inflammation impacts leukemia development, aged wild‐type and IL‐37 transgenic (IL‐37tg) mice were transplanted with BCR‐ABL1^+^/Arf‐null B‐ALL cells (Figure S9A). Due to the presence of a strong driver mutation (BCR‐ABL1) and the lack of a potent tumor suppressor (Arf), these cells are capable of establishing leukemia in mice without myeloablation, which leaves the immune system unperturbed (Boulos et al., 2011; Manlove et al., 2015; Rabe et al., 2019; Williams et al., 2006, 2007). After transplantation into aged wild‐type mice, all mice succumbed to disease within 2 months post‐injection of B‐ALL cells (Figure S9B). The transgenic expression of IL‐37 in aged mice resulted in a significant extension of survival, such that almost half of the mice injected with B‐ALL cells survived for over 2 months (Figure S9B). In summary, these data demonstrate that IL37 expression in aged mice protects against B‐ALL progression.

**FIGURE 5:**
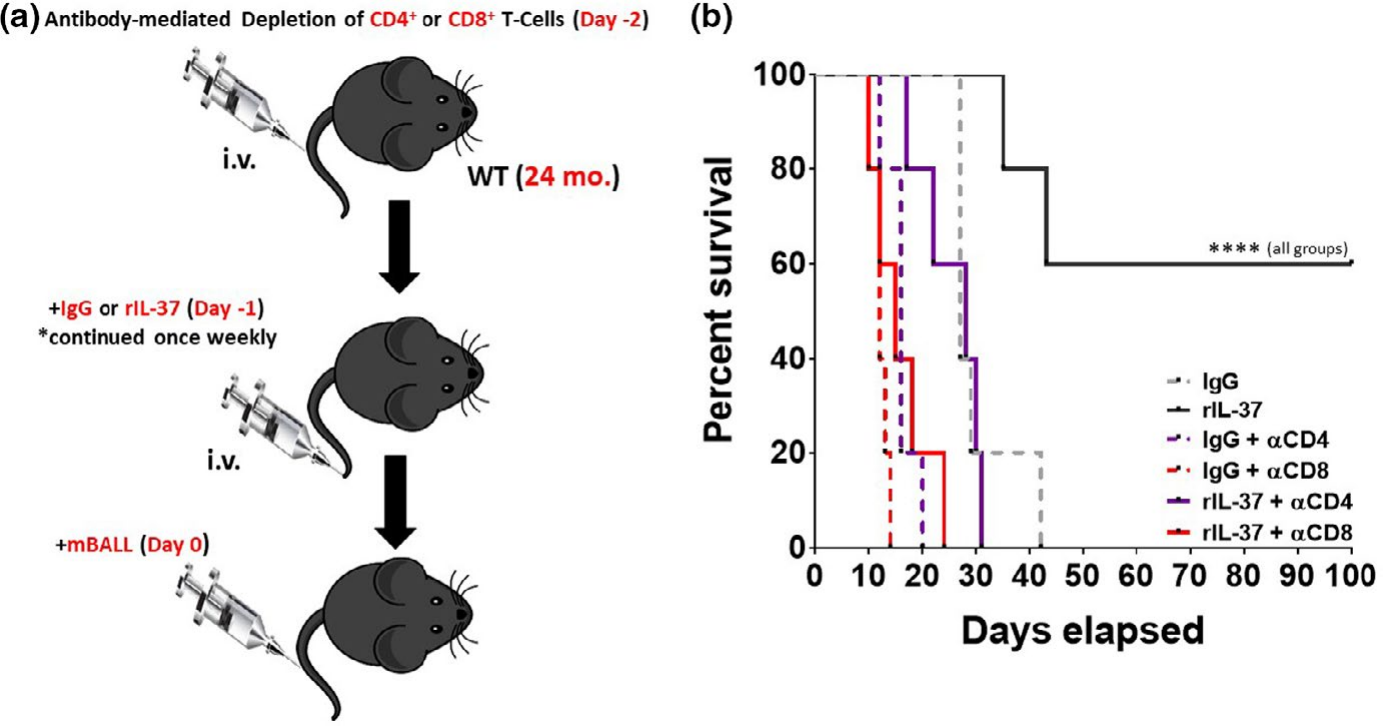
Recombinant IL‐37 treatment protects aged mice from B‐ALL pathogenesis in a T‐cell dependent manner. (a) Aged (24 months old) C57BL/6 mice were treated with T‐cell depleting antibodies (αCD4 and αCD8) 2 days prior to intravenous challenge with BCR‐ABL^+^ *Arf* ^−^ *^/^* ^−^ murine B‐ALL cells (mB‐ALL). Mice were also treated with Control Ig or rIL‐37 1 day prior to the injection of mB‐ALL cells, and this treatment continued throughout the experiment. (b) Survival was monitored for over 3 months. Significance was determined using log‐rank test with ****p < 0.0001 indicating a significant extension of survival in aged mice treated with rIL‐37 relative to each experimental group tested. *n* = 5 mice/group

Recent studies have demonstrated that T‐cells are required for the control of B‐ALL development, which is in part regulated by the pro‐inflammatory microenvironment (Rabe et al., 2019). Given these observations, we next determined whether treating aged mice with rIL‐37 could improve T‐cell‐mediated anti‐leukemia responses. To this end, aged mice were treated with Control Ig or T‐cell depleting antibodies followed by treatment with Control Ig or rIL‐37 prior to injection with BCR‐ABL1^+^/Arf‐null B‐ALL cells (Figure 5a). Mice were treated with control Ig or rIL‐37 for the duration of this experiment.

Given the aggressive nature of this leukemia, all mice succumbed to disease within 42 days post‐injection if left untreated (Figure 5b). Impressively, 60% of mice treated continuously with rIL‐37 exhibited survival for greater than 3 months post‐injection of B‐ALL cells (Figure 5b). This protective effect was abrogated when CD4^+^ and CD8^+^ T‐cells were depleted, suggesting that both T‐cell populations are essential for immunity against B‐ALL cells. In line with recent studies (Rabe et al., 2019), these data confirm the importance of T‐cells in the protection against B‐ALL pathogenesis. Importantly, these data demonstrate that treating aged mice with recombinant IL‐37 significantly boosts anti‐leukemia T‐cell‐mediated immune responses.

### 2.6 Recombinant IL‐37 treatment improves the efficacy of aged chimeric antigen receptor (CAR) T‐cells

**FIGURE 6:**
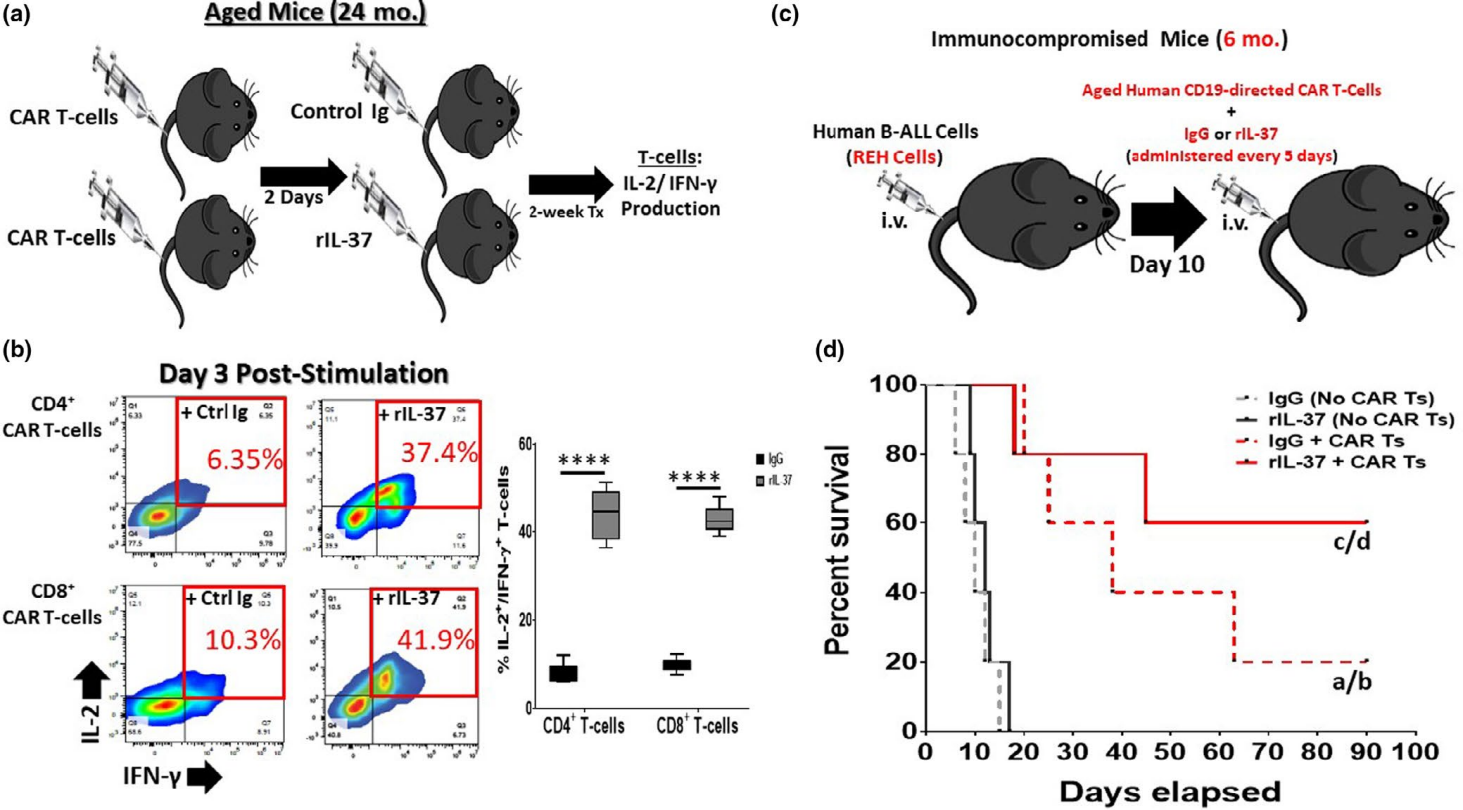
Recombinant IL‐37 Treatment Improves the Efficacy of Aged CAR T‐cells. (a) Murine CD3^+^ T‐cells were purified from aged (24 months old) C57BL/6 wild‐type mice and transduced to express CD19‐directed CARs (transduced cells express GFP). Aged CAR T‐cells were then injected into aged wild‐type mice which were then treated with control immunoglobulin (Control Ig) or recombinant IL‐37 (rIL‐37) once weekly for 2 weeks. After 2 weeks of treatment, GFP^+^ CAR T‐cells were sorted from mice and stimulated in vitro with CD19‐expressing murine BALL cells. On day 3 of culture, IL‐2 and IFN‐γ production from aged CAR T‐cells was assessed by flow cytometric analysis. (b) Representative flow cytometry and quantitative data showing the percentage of IL‐2 and IFN‐γ producing aged CAR T‐cells. Means ± *SD* are shown in (c) with ****p < 0.0001 determined using a Student's *t* test. *n* = 9 mice/group with 3 independent experiments conducted. (c) NOG immunocompromised (6 months old) mice were intravenously challenged with human B‐ALL cells (REH cells). On day 10 post‐transplantation (when signs of morbidity were observed in all mice), mice were injected with CD19‐directed CAR T‐cells from an aged donor (67 years old). Mice were simultaneously injected with Control Ig or rIL‐37 and this treatment was continued every 5 days until the experiment was terminated. (d) Survival was monitored for over 3 months. Significance was determined using log‐rank test with “a” denoting significance between IgG + CAR Ts and IgG (No CAR Ts) treated groups, “b” denoting significance between IgG + CAR Ts and rIL‐37 (No CAR Ts) treated groups, “c” denoting significance between the rIL‐37 + CAR Ts and IgG (No CAR Ts) treated groups, and “d” denoting significance between the rIL‐37 + CAR Ts and rIL‐37 (No CAR Ts) treated groups. *n* = 5 mice/group

Given the ability of recombinant IL‐37 to boost the function of aged T‐cells, we next determined how rIL‐37 treatment altered the efficacy of aged CAR T‐cells. To this end, CD19‐expressing CAR T‐cells were engineered from T‐cells isolated from aged (24 months old) mice and injected into aged (24 months old) recipient mice. On day 2 post‐transplantation, mice were treated once weekly for 2 weeks with Control Ig or rIL‐37. CAR T‐cells were then purified from the spleen and stimulated with murine CD19‐expressing B‐ALL cells to determine the ex vivo production of IL‐2 and IFN‐γ (Figure 6a). Consistent with the improvements in the function of aged endogenous T‐cells, rIL‐37 treatment also increased IL‐2 and IFN‐γ production from aged CD4^+^ and CD8^+^ CAR T‐cells (Figure 6b).

Given the ability of rIL‐37 treatment to augment the function of aged murine CAR T‐cells, we next determined how rIL‐37 treatment impacted the efficacy of aged human CAR T‐cells in vivo. Immunocompromised mice (6 months old) were transplanted with human B‐ALL cells, and all mice injected with B‐ALL cells exhibited signs of morbidity by day 7 post‐transplantation (data not shown). On day 10 post‐transplantation of B‐ALL cells, mice began receiving treatment with human CD19‐directed CAR T‐cells (generated from a 67‐year‐old donor; Figure S10) with or without the coadministration of rIL‐37 (which continued weekly for the duration of the experiment; Figure 6c). In these experiments, we found that treating mice with human CAR T‐cells and control Ig resulted in 20% of mice surviving for greater than 3 months (Figure 6d). When CAR T‐cell therapy was combined with rIL‐37 treatment, the 3‐month survival of mice significantly increased to 60% (Figure 6d).

Overall, the results of our study demonstrate that IL‐37 can rejuvenate the function of aged endogenous T‐cells and boost the efficacy of aged CAR T‐cells resulting in attenuated B‐ALL pathogenesis.

## 3 DISCUSSION

We have previously reported that transgenic IL‐37 expression in aged mice rejuvenated the function of aged B‐progenitor cells and abrogated the selection of B‐cells harboring oncogenic mutations; thereby, preventing leukemogenesis (Henry et al., 2015). In this study, we delineated how IL‐37 impacted the function of mature immune cells.

Notably, we found that the IL‐37 gene expression levels were significantly lower in monocytes isolated from donors 65 years of age or older relative to their younger counterparts, suggesting that inflammaging is accompanied by lower levels of IL‐37 production from innate immune cells. This observation is consistent with published data suggesting that IL‐37 gene expression levels are lower in the diseased synovia of patients with rheumatoid arthritis and other inflammatory diseases including allergic rhinitis, asthma, and non‐small cell lung cancer (Cavalli et al., 2016).

Furthermore, we demonstrate for the first time, to our knowledge, that treating aged mice with recombinant IL‐37 abrogates aging‐associated splenomegaly. This change was accompanied by restoring a youthful balance of CD4^+^ to CD8^+^ T‐cells and evoking youthful gene expression programs in T‐lymphocytes. Of particular interest, the gene expression levels of the linker for the activation of T‐cells (*Lat*) was found to be increased to youthful levels in aged CD4^+^ and CD8^+^ T‐cells recovered from old mice receiving rIL‐37 treatment. This observation suggests that rIL‐37 treatment augments TCR‐mediated signaling in aged T‐cells. Indeed, T‐cells isolated from aged mice receiving rIL‐37 responded more robustly to αCD3/αCD28 stimulation which mimics peptide‐MHC/TCR activation. Indeed, both T‐helper cells and cytotoxic lymphocytes exhibited significantly enhanced IL‐2 and IFN‐γ production with this mode of stimulation. Recombinant IL37 treatment of aged mice also significantly reduced *Pdcd1* (the gene encoding for PD‐1) and significantly increased Stat4 gene expression levels in T‐helper cells, suggesting an attenuation of T‐cell exhaustion and enhanced IL‐12‐mediated signaling (which may play a role in augmenting IFN‐γ production from aged T‐cells). In addition to modifying gene expression profiles, rIL‐37 treatment of aged mice resulted in decreased surface expression of the immunosuppressive molecules PD‐1, Tim‐3, and TIGIT on activated T‐cells coincident with increased proliferation after in vitro stimulation. The increase in the proliferation in aged T‐cell is notable, because aged microenvironments are capable of potently suppressing the proliferation of young and aged T‐cells (Quinn et al., 2018). Given that aging‐associated T‐cell dysfunction has been attributed to increased levels of PD‐1, Tim‐3, and TIGIT (Lee et al., 2016; Song et al., 2018), our results suggest that IL‐37‐mediated rejuvenation of aged T‐cells is partially attributed to its ability to downregulate the surface expression of these immunoinhibitory proteins on T‐lymphocytes. In all, these results demonstrate that IL‐37 treatment reprograms gene expression profiles in aged T‐cells resulting in more robust effector functions and an increased threshold for T‐cell exhaustion post‐stimulation.

The ability of IL‐37 to restore youthful gene expression profiles, mitigate immunosuppressive mechanisms, and enhance effector T‐cell function is attributed to both direct effects on T‐cells and modulation of the immune environment. We found that transgenic expression of IL‐37 and rIL‐37 treatment attenuated aging‐associated increases in circulating IL‐1β, IL‐6, and TNF‐α levels. Given that chronic TNF‐α exposure suppresses T‐cell receptor signaling (Cope et al., 1997), blocking TNF‐α enhances CD8^+^ T‐cell responses in murine models of melanoma (Bertrand et al., 2015), and TNF‐α/PD‐1 gene expression levels are positively correlated in patients with melanoma (Bertrand et al., 2017), we determined whether IL‐37 antagonized TNF‐α signaling in aged T‐cells. In immune cells, TNF‐α stimulation potently activates NF‐κB, which has multiple binding sites in the T‐cell PD‐1 promoter region (Redd et al., 2018). We found rIL‐37 directly antagonized TNF‐α‐mediated NF‐κB activation, which is consistent with published observations demonstrating similar effects in other pathological settings (Cavalli & Dinarello, 2018; Li et al., 2017; Nold et al., 2010; Xie et al., 2016). Furthermore, IL‐37 treatment significantly reduced PD‐1 surface expression and genes activated downstream of both PD1 and TNF‐α signaling pathways (TMEM16F, GM130, PD‐1, and SHP2). Directly stimulating aged T‐cells with rIL‐37 also augmented the expression levels of genes which promote interferon production (IFN‐γ, TBK1, and IRF3), coincident with increased IFN‐γ production from aged T‐cells after αCD3/αCD28 stimulation. Our observations support recent studies demonstrating that IL‐37 treatment restores normal T‐cell function (reduction in IL‐17 production) in the chronic inflammatory setting of allergic rhinitis (Li, Shen, et al., 2018).

In addition to IL‐37‐mediated cell autonomous changes in aged T‐cells, aging‐associated increases in myelopoiesis were abrogated after treating aged mice with rIL‐37. Similarly, splenic DC and macrophage populations were also decreased to youthful levels after aged mice received rIL‐37 treatment. The reduction in myeloid cells in the bone marrow and spleens of aged mice treated with rIL‐37 likely contributed to the significantly lower levels of circulating pro‐inflammatory cytokines and more robust T‐cell effector functions. Mechanistically, IL‐37 binds the IL‐18Rα and IL‐1R8 receptors, which are expressed on myeloid cells and T‐cells, and attenuates the production of pro‐inflammatory cytokines by inhibiting transforming growth‐factor‐β‐activated protein kinase 1 (TAK1), NF‐κB, and MAPK activity (Cavalli & Dinarello, 2018; Lunding et al., 2015; Nold et al., 2010). The “renormalization” of the inflammatory microenvironment in aged mice which we demonstrate in this study is consistent with reported protective effects of IL‐37 in other pathological inflammatory settings including endotoxin shock syndrome, lung and spinal cord injury, colitis, coronary artery disease, and arthritis (Cavalli & Dinarello, 2018).

Aging in mice and humans is associated with extensive immunological change including the onset of chronic inflammation and the development of compromised T‐cell‐mediated immunity (Henry et al., 2011; Ponnappan & Ponnappan, 2011; Ventura et al., 2017). Augmented immunosuppressive mechanisms in aged individuals are postulated to contribute to increased pathogenic infections and higher cancer incidence which are hallmarks of aging (Ladomersky et al., 2019; Rea et al., 2018). In addition to elevated cancer incidence, cancer‐related mortality rates are significantly higher in older patients (White et al., 2014; Yancik, 2005). The failure to achieve similar survival outcomes in younger and older patients with cancer has been partially attributed to the inability to achieve effective chemotherapy dosages in older patients due to toxicity complications (Repetto, 2003). Given that chemotherapies are less effective in older patients (Kim & Hurria, 2013; Repetto, 2003), other therapeutic options, such as treatments using immunotherapies, are beginning to be used to treat older patients with solid and hematological malignancies. Indeed, CAR T‐cell therapy is currently being used to treat relapsed and refractory B‐ALL and DLBCL with new clinical trials open to test the efficacy of this cell‐based therapy as a frontline option (Chavez et al., 2019; Hay & Turtle, 2017). Despite the success of CAR T‐cell therapy, between 20 and 50 percent of the pediatric and adult patients receiving this form of immunotherapy will relapse within 2 years of treatment (Cao et al., 2018; Gardner et al., 2017; Lee et al., 2015; Li, Zhang, et al., 2018; Maude, Frey, et al., 2014; Maude et al., 2018; Park et al., 2018; Turtle et al., 2016; Xu et al., 2019). The failure to achieve durable responses in patients receiving CAR T‐cell therapy has resulted from receiving low potency CAR T‐cells and the loss of target antigens on cancer cells (Xu et al., 2019). Additional studies in animal models and patients will be required to identify additional mechanisms of immune evasion.

In laboratory settings, the importance of using appropriate model systems for the pre‐clinical development and validation of immunotherapies is paramount for efficacy and safety testing prior to clinical trials (Bouchlaka & Murphy, 2013; Repetto & Balducci, 2002). The incidence of most leukemias rises dramatically in individuals over 65, and mortality rates are higher in geriatric patients (Repetto & Balducci, 2002). Despite the strong association between aging and leukemia development, most of the pre‐clinical studies of immunotherapies are conducted in young mice (Bouchlaka & Murphy, 2013; Repetto & Balducci, 2002). This is concerning given that a major hallmark of aging in mice and humans is attenuated immune function (Fane & Weeraratna, 2020). The immune microenvironment “edits” cancer cells, and these changes dictate tumor cell eradication, equilibrium, or immune escape (Gonzalez et al., 2018). Given the impact of the immune microenvironment on cancer progression and the immunological decline associated with aging (Gonzalez et al., 2018), there is a growing need to study how immunotherapies behave (efficacy and toxicity) in aged recipients.

Given the lack of preclinical studies and scant clinical data regarding the efficacy of CAR T‐cell therapy in patients over 65 (van Holstein et al., 2019), our study is the first to demonstrate that functional defects in aged endogenous T‐cells are transferable to engineered T‐cells and are not completely overcome by the introduction of a CAR. Our findings corroborate a recent study demonstrating age‐dependent functional defects in CAR T‐cells engineered from old (>65 years old) relative to young (18–45 years old) donors (Guha et al., 2017). In murine studies, we demonstrate that aging‐associated increases in chronic inflammation, the onset of splenomegaly, and the accumulation of myeloid populations in the bone marrow and spleen can be prevented by the anti‐inflammatory cytokine IL‐37. Importantly, we demonstrate that treating aged mice with rIL‐37 reduces TNF‐α signaling and significantly decreased the surface expression of PD‐1 on naïve CD4^+^ and CD8^+^ T‐cells. This effect was not limited to endogenous T‐cells, as demonstrated by the results that rIL‐37 treatment also prevented high PD‐1 surface expression on aged CAR T‐cells. Impressively, the function of endogenous and CAR T‐cells was improved by rIL‐37 treatment, leading to increased cytokine production *ex vivo* and the augmented protection of mice with B‐ALL.

Our study highlights the potency of recombinant IL‐37 treatment in boosting T‐cell‐mediated immunity in aged backgrounds and its ability to increase the efficacy of aged CAR T‐cells. Importantly, our results demonstrate that components of aging‐associated immune senescence are reversible and further support emerging literature demonstrating the utility of targeting the inflammatory microenvironment as a viable option to improve the efficacy of immunotherapies (Bouchlaka et al., 2013; Maude, Barrett, et al., 2014).

## 4 EXPERIMENTAL PROCEDURES

### 4.1 Mice

BALB/c and C57BL/6 mice of different ages were purchased from the National Institute of Aging (NIA) or the National Cancer Institute (NCI) and were used for all experiments in this study with the expectation of data presented in Figure 6c,d. Interleukin‐37 (IL‐37) transgenic mice were backcrossed onto a C57BL/6 background for more than 10 generations (Nold et al., 2010), and both transgene‐positive and transgene‐negative littermates were aged in‐house. CIEA NOG immunodeficient mice (nomenclature: NOD.Cg‐ *Prkdc^scid^Il2rg^tm1Sug^*/JicTac) were purchased from Taconic Biosciences and maintained in‐house. These mice were used for xenograft experiments presented in Figure 6C,D. Female and male mice were used in these studies.

### 4.2 Cell lines

Human and murine B‐cell acute lymphoblastic leukemia (B‐ALL) cell lines were gifted from the laboratories of Dr. Douglas Graham and Dr. Christopher Porter (Department of Pediatrics; Emory University School of Medicine). Human B‐ALL cells (REH) and murine B‐ALL cells (GFP‐expressing, BCR‐ABL1^+^/Arf‐null) were grown in RPMI 1640 media supplemented with 20% FBS.

### 4.3 Construction of the CD19‐CAR

The sequence for the CD19‐directed single chain variable fragment (scFv) was generated using the published anti‐CD19 murine immunoglobulin protein sequence (FMC63) (Nicholson et al., 1997), and the cDNA sequence designed to express the scFv was codon optimized for optimal expression in human cells using the codon optimization tool from IDT (Coralville, IA). The C‐terminus of V_H_ was joined with the N‐terminus of V_L_ using a 15 bp linker encoding a glycine and serine pentapeptide repeat (G4S)_3_ (Huston et al., 1993). The gene block for the CD19 scFv cDNA sequence was created by Genewiz (South Plainfield, NJ). The CD19 scFv sequence was then cloned into the CAR of our cassette, which is a second‐generation CAR consisting of the transmembrane and intracellular domains of CD28, and the intracellular signaling domain of CD3z (Raikar et al., 2018). A CD8 hinge region connects the CD19 scFv to the CD28 domain. A bicistronic vector coexpressing eGFP and the CD19‐CAR via a self‐cleaving 2A peptide sequence (P2A) was used to enable identification of positively transduced cells by flow cytometry.

### 4.4 T‐cell activation assays

#### 4.4.1 Endogenous T‐cell assays

CD4^+^ or CD8^+^ T‐cells were purified from aged mice using Magnetic‐activated cell sorting (MACs) as described above. T‐cells were stimulated (10^4^–5 × 10^4^ cells/well) in 96‐well flat bottom plates (Millipore Sigma; cat. no. CLS3997) coated with αCD3 (10 µg/ml; BD Biosciences; cat. no. 553057) and αCD28 (2 µg/ml; BD Biosciences; cat. no. 553294) antibodies in RPMI 1640 media supplemented with 10% FBS. On day 3 of culture, T‐cells were harvested, and intracellular cytokine staining was performed as previously described (Henry et al., 2008, 2010) to determine interleukin‐2 (αIL‐2 PE; Biolegend; cat. no. 503808) and interferon‐gamma (αIFN‐γ APC; Biolegend; cat. No. 505810) production using flow cytometry (% positive and mean fluorescence intensity [MFI]). To determine CD44 and PD‐1 surface expression on murine T‐cells, cells were surface stained with αPD‐1 PE (Biolegend; cat. no. 135205) and αCD44 APC (Biolegend; cat. no. 103012) for 1 h (covered on ice) and the MFI for each marker was determined using flow cytometry. All flow cytometry data were analyzed using the FlowJo software (BD Biosciences).

#### 4.4.2 CAR T‐cell assays

GFP‐expressing murine CD19‐directed CAR T‐cells were harvested from aged mice after adoptive transfer using Fluorescence‐activated cell sorting (FACs). Murine CAR T‐cells were stimulated *ex vivo* with murine CD19‐expressing BCR‐ABL1^+^/Arf‐null B‐ALL cells. On day 3 of culture, cells were harvested and surface stained for 1 h (covered on ice) with αCD4 Pacific Blue (Biolegend; cat. no. 100428) or αCD8 Pacific Blue (Biolegend; cat. no. 100725). Following surface staining, intracellular cytokine staining for IL‐2 and IFN‐γ production was performed as described above.

### 4.5 Murine experiments

#### 4.5.1 T‐cell depletion experiments

T‐cells were depleted from aged (24 months) C57BL/6 mice using CD4 (anti‐CD4; clone GK1.5; purchased from Bio X Cell) and CD8 (anti‐CD8α; clone 53–6.72 and anti‐CD8β; clone 53–5.8; purchased from Bio X Cell) T‐cell depleting antibodies (i.p.; 0.5 mg/mouse/each antibody for two consecutive days). One day after the administration of T‐cell depleting antibodies, mice were treated with Control Ig (i.v.; 100 µg/mouse) or rIL‐37 (i.v.; 100 µg/mouse), and these treatments continued once weekly for the duration of the experiment. Two days after the administration of T‐cell depleting antibodies, mice were transplanted with murine B‐ALL (GFP‐expressing, BCR‐ABL1^+^/Arfnull) cells (i.v.; 2 × 10^4^ B‐ALL cells/ mouse) and survival was monitored for >3 months. Once signs of leukemia manifested (the detection of GFP^+^ cells in the peripheral blood, lethargy, ruffled fur, labored breathing, or greater than 7% weight loss), mice were removed from the study.

#### 4.5.2 CAR T‐cell adoptive transfer experiments

Aged mice were conditioned with busulfan (i.v.; 25 mg/kg; (Henry et al., 2015)) and injected with GFP‐expressing, murine CD19‐directed CAR T‐cells (i.v.; 10^6^ cells/mouse) on day 2 post‐conditioning. Mice were treated with Control Ig (i.v.; 100 µg/mouse) or rIL‐37 (i.v.; 100 µg/mouse) beginning on day 2 post‐adoptive transfer of CAR T‐cells and continued receiving treatment once weekly for 2 weeks. After the 2‐week treatment period, GFP‐expressing CAR T‐cells were harvested for functional analysis as described above.

#### 4.5.3 Xenograft studies

Male and Female CIEA NOG Mice (immunodeficient mice lacking T‐, B‐, and NK cells) were transplanted intravenously (i.v.) with 5 × 10^4^ CD19‐expressing human B‐ALL cells (REH cells). After signs of morbidity were observed (day 7), mice were treated with human CD19‐directed CAR T‐cells (i.v.; 10^6^/mouse) from a 67‐year‐old donor beginning on day 10 post‐transplantation of human B‐ALL cells. Mice were simultaneously treated with Control Ig or rIL‐37 (i.v.; 100 µg/mouse/each group), and this treatment was continued (without co‐administration of additional CAR T‐cells) once weekly for the duration of the experiment in surviving mice. Mice were removed from the study when leukemia‐induced signs of morbidity manifested (lethargy, ruffled fur, or labored breathing).

All animal experiments were approved by and performed in accordance with guidelines of the IACUC of the University of Colorado Anschutz Medical Campus and Emory University School of Medicine.

### 4.6 Statistics

Unpaired *t* tests, Cox proportional hazards tests, and one‐way ANOVA were used to analyze the data, with a *p*‐value of less than 0.05 considered statistically significant. All error bars represent biological replicates, not technical replicates. Statistical analyses were performed with GraphPad Prism software, version 8.4.2 (GraphPad Software). All results are expressed as the mean ± SEM.

## CONFLICT OF INTEREST

The authors declare that they have no conflicts of interest with the contents of this article.

## AUTHOR CONTRIBUTIONS

JAH collected and interpreted data, created the manuscript figures, provided financial support, and assisted with drafting the manuscript. MYL assisted with each experiment including the qPCR analyses. RH assisted with in vivo experiments, qPCR analyses, and data interpretation. RSA assisted with in vivo experiments and T‐cell purification. JS generated CAR T‐cells. GT assisted with murine experiments. TS assisted JAH with graphical abstract design and data interpretation. DBB assisted with rIL‐37 experiments. AF generated all CAR T‐cells for this study. CPP provided Bcr‐Abl^+^/Arf‐null murine B‐ALL cells, interpreted data, and reviewed the manuscript. EEZ created, synthesized, and provided recombinant IL‐37 for these studies. CAD provided IL‐37 transgenic mice, proposed study designs, provided financial support, and reviewed the manuscript. SSR generated CAR T‐cells, assisted with experimental design, provided financial support, and contributed to the drafting of this manuscript. JD provided financial support for the study, interpreted data, and reviewed the manuscript. CJH conceived the study, provided financial support for the study, performed experiments, analyzed data, and drafted the manuscript. All authors read and approved the final manuscript.

## Supporting information

Fig S1‐S10

Supplementary Material

## Data Availability

The data that support the findings of this study are available from the corresponding author upon reasonable request.
