# Supplementary material for: Interleukin‐37 improves T‐cell‐mediated immunity and chimeric antigen receptor T‐cell therapy in aged backgrounds
Source: Aging Cell. 2021 Jan 22;20(2):e13309. doi: 10.1111/acel.13309 (PMC7884049; doi:10.1111/acel.13309)
Supplement: Supplementary file 1 — Fig S1‐S10 [file ACEL-20-e13309-s001.pdf]

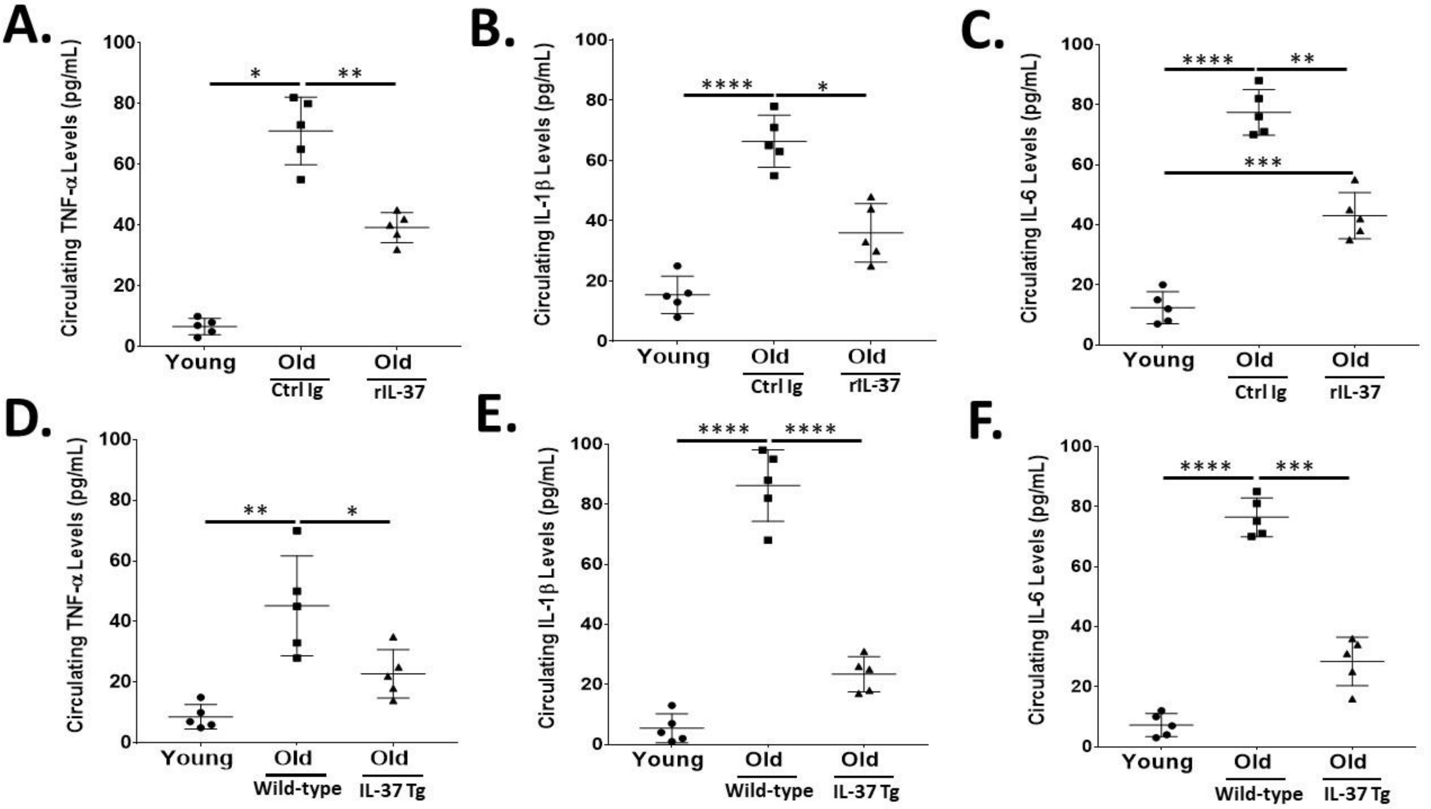

Figure S1. **Transgenic Expression of and Treating Aged Mice with Interleukin-37 Abrogates Aging-associated Chronic Inflammation**. (*A-C*) Aged (24 months old) mice were treated with Ctrl Ig and rIL-37 intravenously every 2 days for 2 weeks. Serum was collected from mice and circulating levels of TNF-α, IL-1β, and IL-6 were determined via ELISA analysis. (*D-F*) Serum was collected from aged (24 months old) wild-type and IL-37tg mice, and ELISA analysis was performed to determine the circulating levels of TNF-α, IL-1β, and IL-6. Means ± s.d. are shown with **p*<0.05, ***p*<0.01, ****p*<0.001, and *****p*<0.0001 determined using a one-way ANOVA with Tukey's post-test. n=5 mice/group.

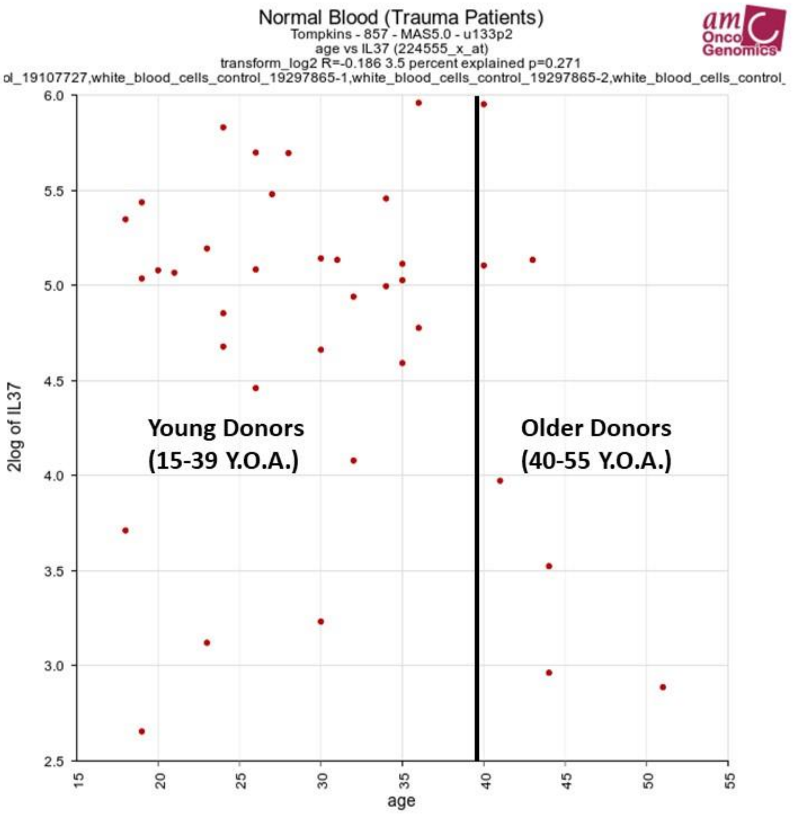

Figure S2. ***IL-37* gene expression levels in leukocytes isolated from young and middle-aged healthy donors.** The R2 Database was utilized to determine the gene expression levels of *IL-37* across age ranges. The database used was from Tompkins study, where 37 healthy control samples were used to determine the gene expression levels of *IL-37* in leukocytes. The GEO ID of the Tompkins study is gse36809.

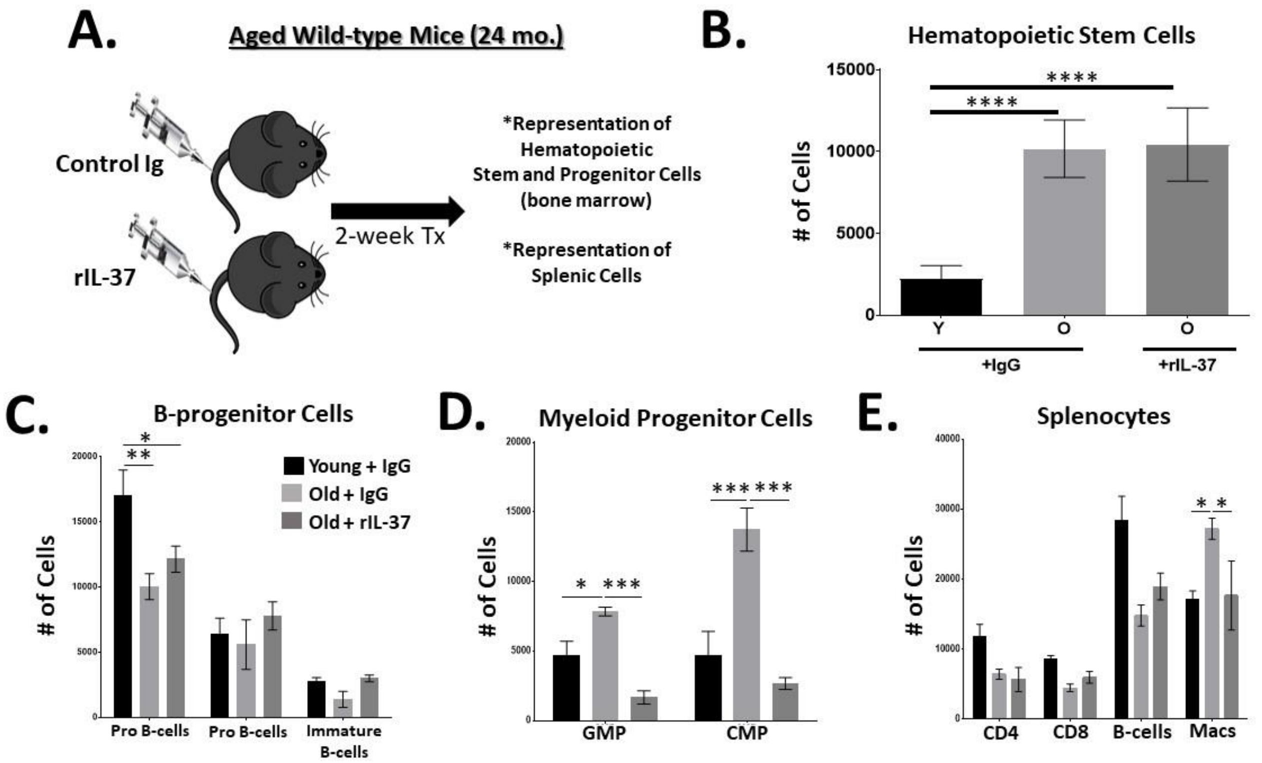

Figure S3. **Treating aged mice with recombinant Interleukin-37 suppresses aging-associated increases in myelopoiesis.** (*A*) Aged (24 months old) C57BL/6 wild-type mice were treated with control immunoglobulin (Control Ig) or recombinant IL-37 (rIL-37) every other day for 2 weeks to assess global changes in immunity. (*B-D*) Bone marrow was isolated from sacrificed mice to determine the representation of hematopoietic stem cells, B-progenitor cells, and myeloid progenitor cells [GMP: Granulocyte-monocyte progenitors/CMP: Common myeloid progenitors] using flow cytometric analysis. (*E*) Spleens were isolated to determine the representation of T-cells ($CD4^+$ and $CD8^+$), B-cells, and macrophages (Macs) using flow cytometric analysis. Means ± s.d. are shown with **p*<0.05, ***p*<0.01, ****p*<0.001, and *****p*<0.0001 determined using a one-way ANOVA with Tukey's post-test in *B-E*. n=5 mice/group.

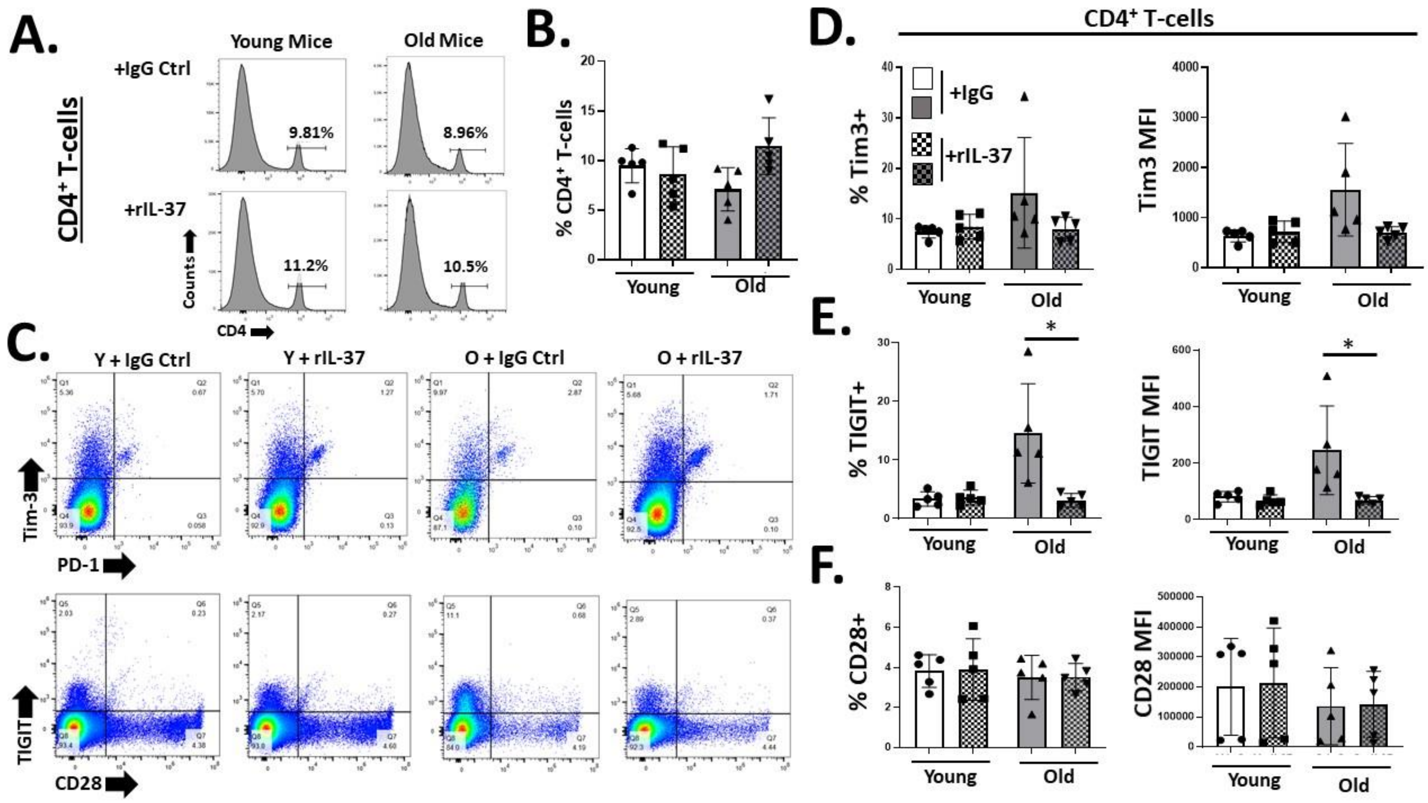

Figure S4. **Recombinant IL-37 treatment of aged mice reduces TIGIT expression on naïve CD4⁺ T-cells.** Aged (24 months old) C57BL/6 wild-type mice were treated with control immunoglobulin (Control Ig) or recombinant IL-37 (rIL-37) every other day for 2 weeks to assess global changes in immunity. (*A*-*B*) Splenocytes were harvested from treated mice, and stained to enumerate the percent of T-helper cells via flow cytometric analysis. (*C*-*F*) Naïve Thelper cells were also stained to assess their surface expression of immunoinhibitory (Tim-3, PD-1, and TIGIT) and costimulatory (CD28) receptors. The percent and MFI of these receptors in *D*-*F* are shown as means ± s.d. with **p*<0.05. Significance was determined using the Student's t-test relative to young + IgG or old + IgG controls with n=5 mice/group.

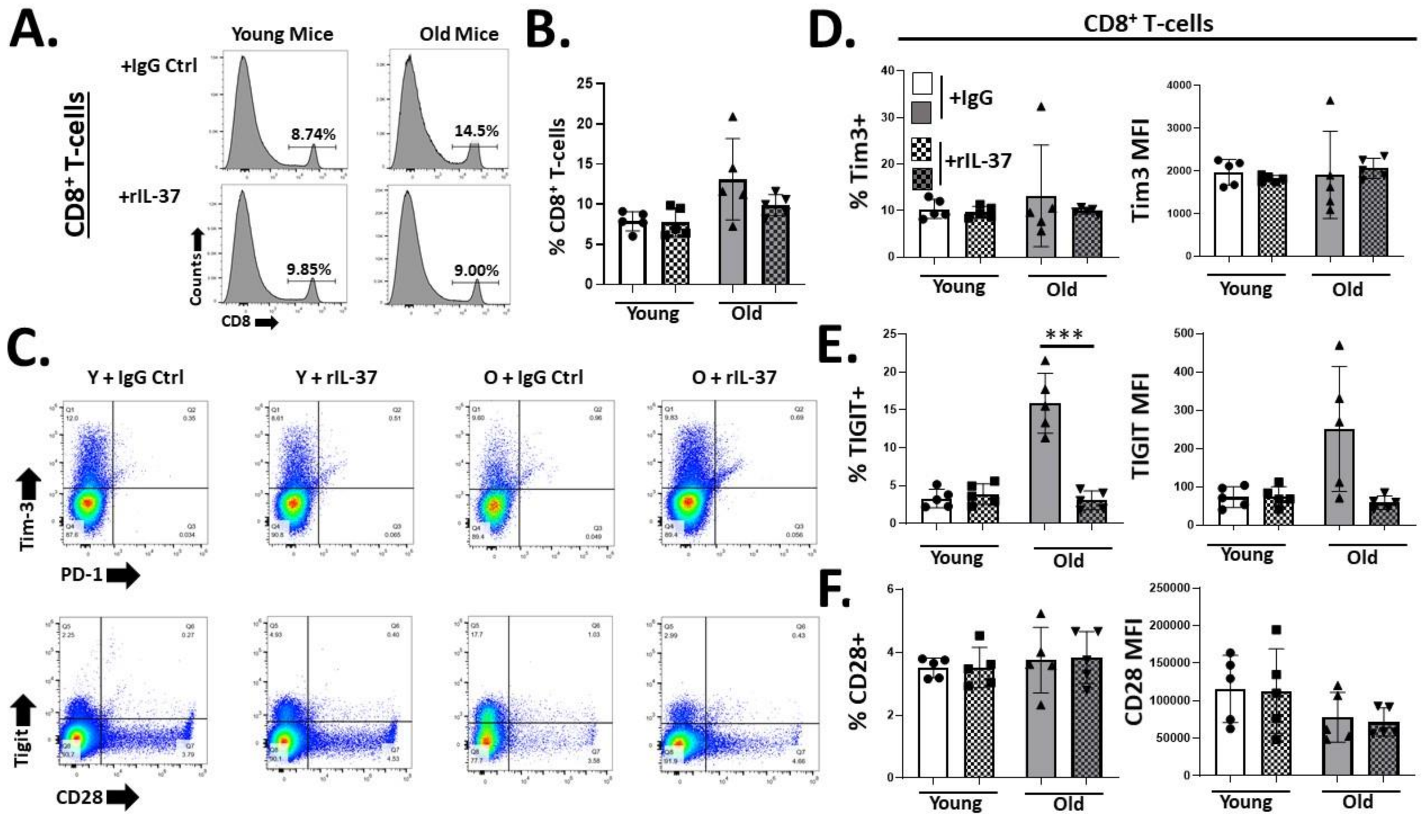

Figure S5. **Recombinant IL-37 treatment of aged mice reduces TIGIT expression on naïve CD8^{+} T-cells.** Aged (24 months old) C57BL/6 wild-type mice were treated with control immunoglobulin (Control Ig) or recombinant IL-37 (rIL-37) every other day for 2 weeks to assess global changes in immunity. (*A-B*) Splenocytes were harvested from treated mice, and stained to enumerate the percent of cytotoxic lymphocytes cells via flow cytometric analysis.. (*C-F*) Naïve cytotoxic lymphocytes were also stained to assess their surface expression of immunoinhibitory (Tim-3, PD-1, and TIGIT) and costimulatory (CD28) receptors. The percent and MFI of these receptors in *D-F* are shown as means ± s.d. with **p*<0.05. Significance was determined using the Student's t-test relative to young + IgG or old + IgG controls with n=5 mice/group.

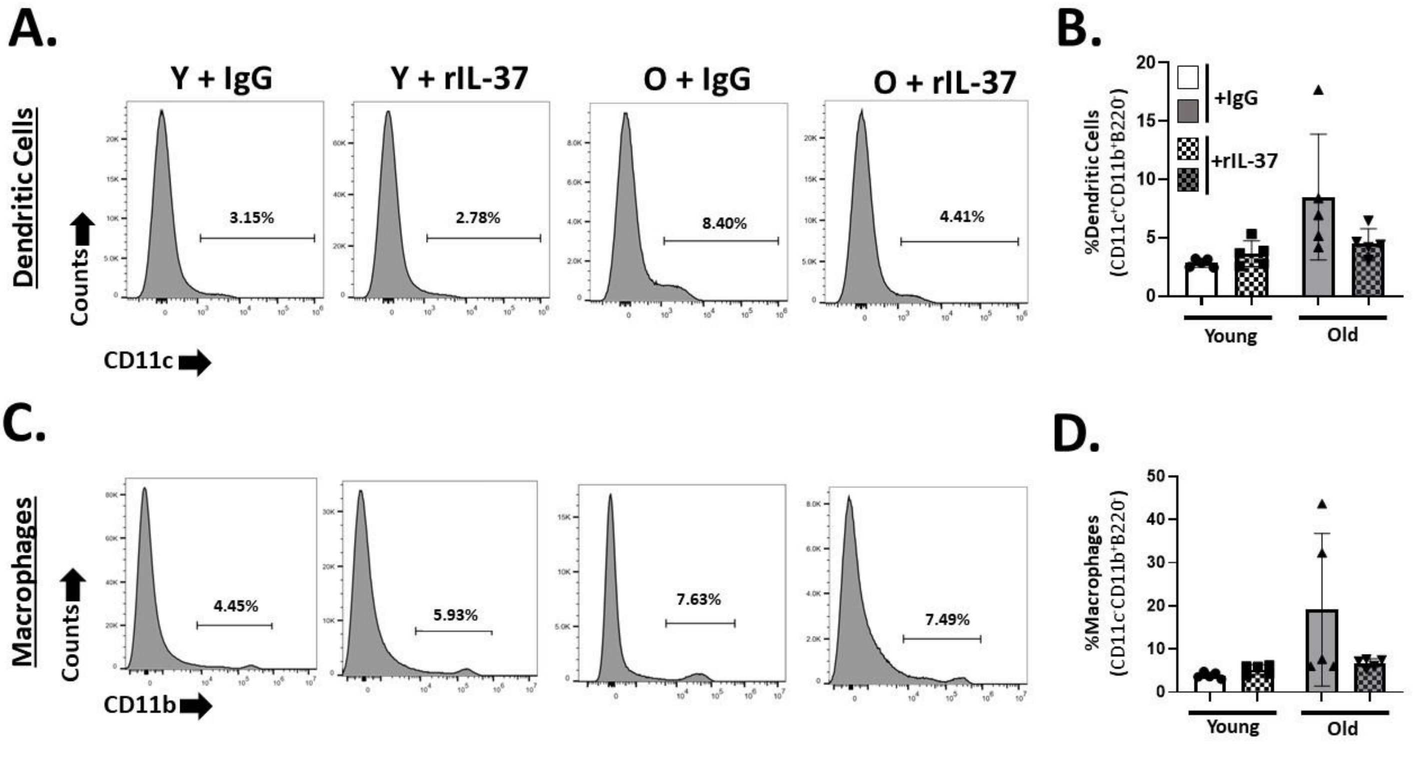

Figure S6. **Recombinant IL-37 treatment of aged mice reduces the percentage of splenic-derived myeloid cells.** Aged (24 months old) C57BL/6 wild-type mice were treated with control immunoglobulin (Control Ig) or recombinant IL-37 (rIL-37) every other day for 2 weeks to assess global changes in immunity. (*A*-*B*) Splenocytes were harvested from treated mice, and stained to enumerate the percent of conventional dendritic cells (CD11c$^+$/CD11b$^+$/B220$^-$, *A* and *B*) and macrophages (CD11c$^-$/CD11b$^+$/B220$^-$, *C and D*) via flow cytometric analysis. The means ± s.d. are shown in *B* and *D* with n=5 mice/group.

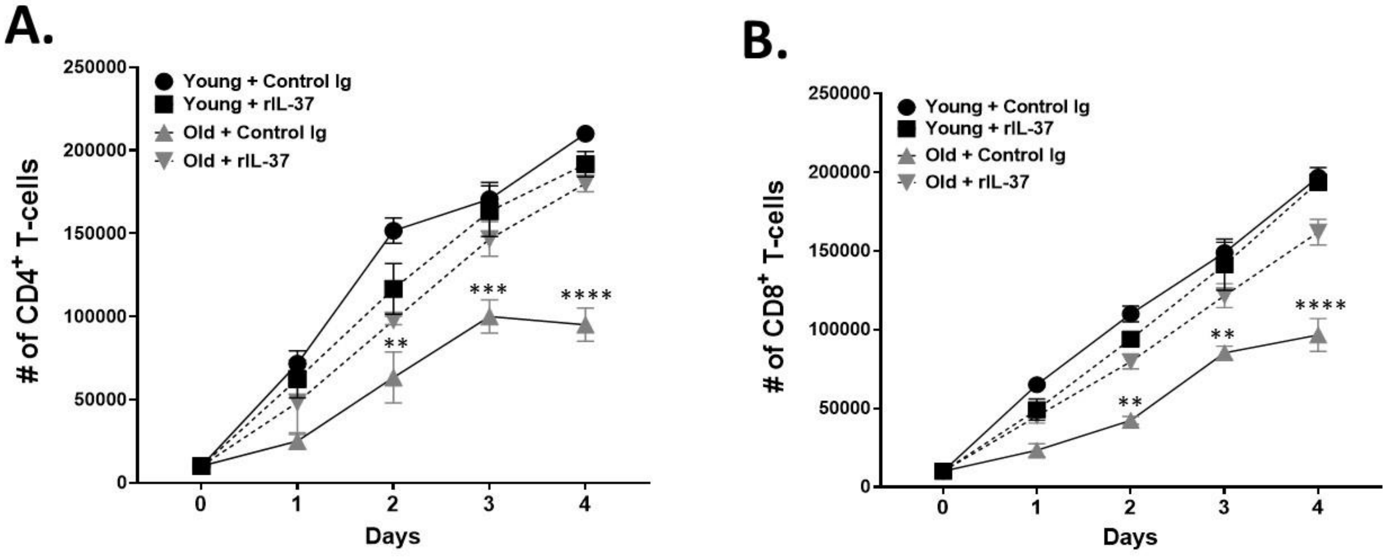

Figure S7. **Recombinant IL-37 treatment abrogates aged T-cell exhaustion leading to a youthful proliferative capacity.** Aged (24 months old) C57BL/6 wild-type mice were treated with control immunoglobulin (Control Ig) or recombinant IL-37 (rIL-37) every other day for 2 weeks. Purified T-cells were stimulated *ex vivo* with αCD3/αCD28 antibody stimulation. T-cells were plated at 2 x 10^4 cells/well on Day 0, and the total number of cells were enumerated each day for 4 days via trypan blue exclusion assays. The means ± s.d. are shown for each time point. Significance was determined using the Student's t-test relative to young + control Ig group with n=5 mice/group.

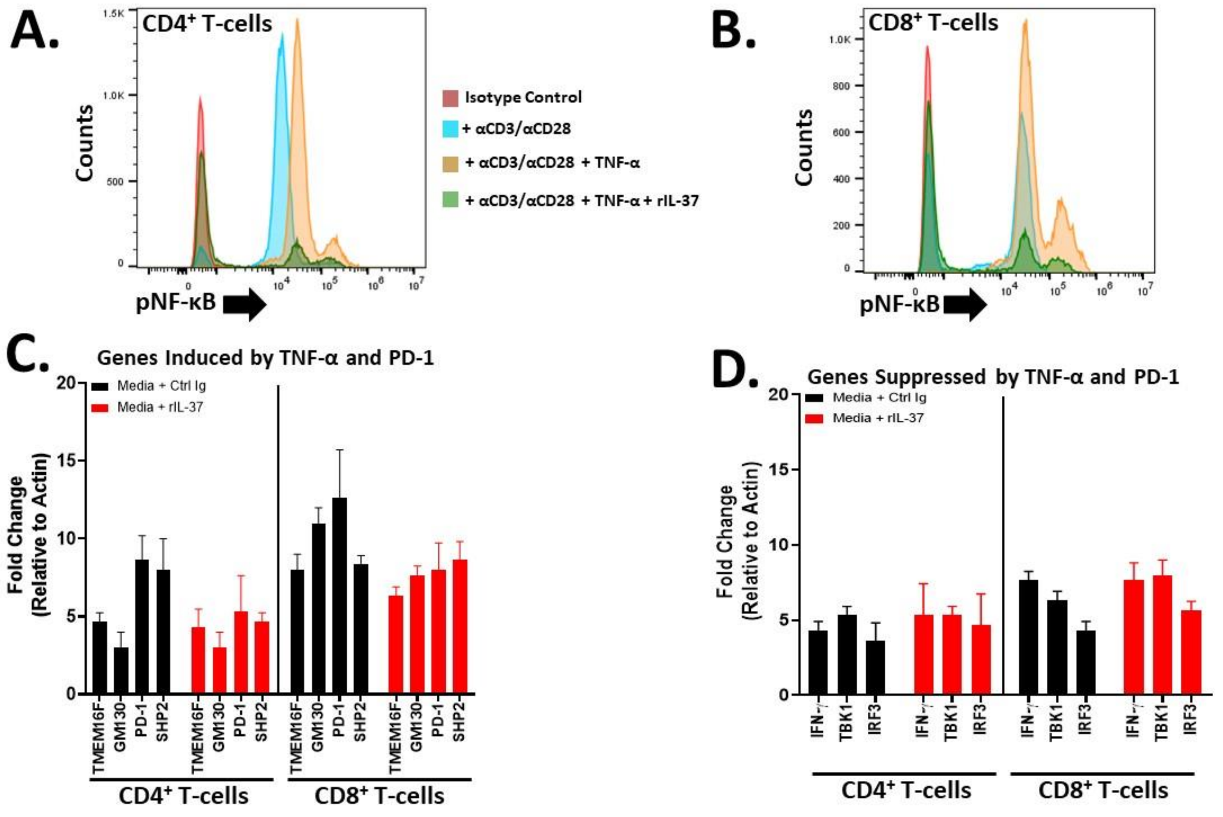

Figure S8. **Recombinant IL-37 treatment opposes TNF-α signaling in aged, but not young, T-cells.** Naïve CD4$^+$ and CD8$^+$ T-cells were purified from aged (24 months old) C57BL/6 mice via MACs selection and stimulated *in vitro* with αCD3/αCD28 in the presence Control Ig, rTNF-α, or rTNF-α + rIL-37. (*A* and *B*) After 10' of stimulation, phospho-flow cytometry was performed to determine NF-κB activation. Representative data are shown. (*C* and D) The experiment described above was performed on naïve T-cells purified from young (2 months old) C57BL/6 mice. Naïve T-cells were stimulated with Control Ig or rIL-37 for 4 hours. After the stimulation period, qPCR analysis was performed to ascertain the expression levels of genes involved in T-cell activation (IFN-γ, TBK1, IRF3) and inhibition (TMEM16F, GM130, SHP2, and PD1). Means + s.d. are shown with n=9 mice/group (3 independent experiments were conducted).

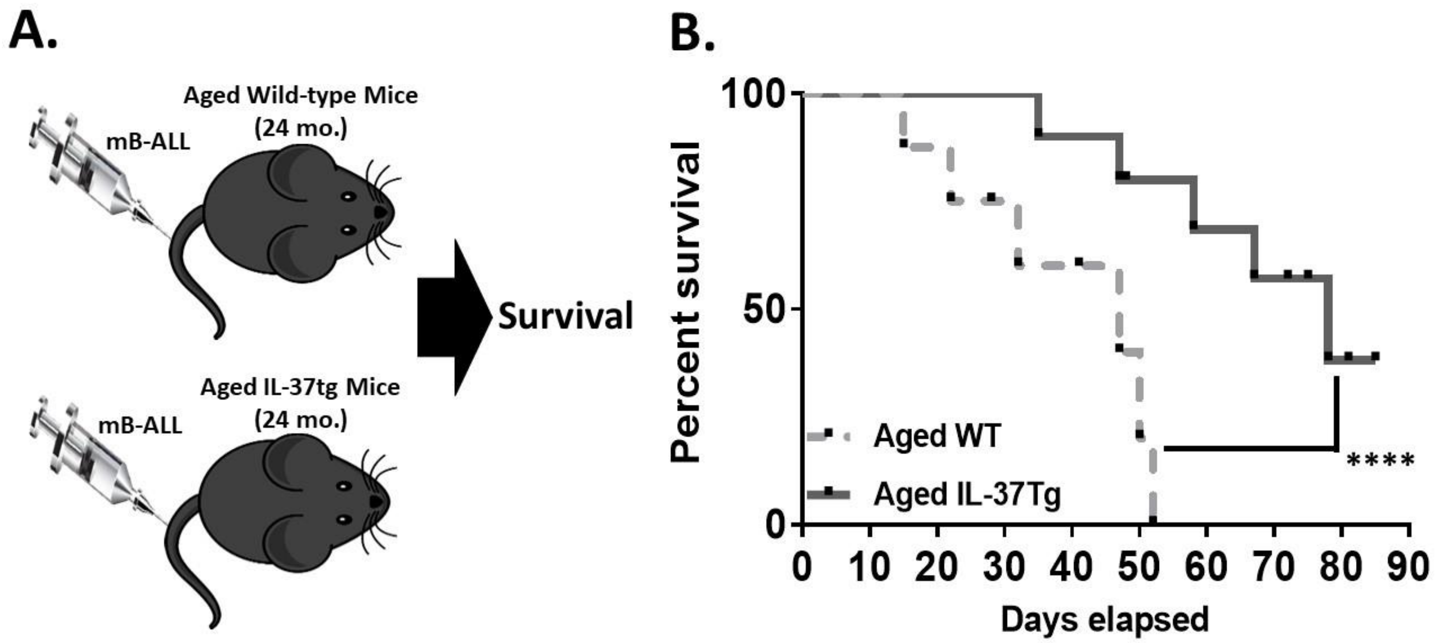

Figure S9. **Interleukin-37 protects against B-cell acute lymphoblastic leukemia pathogenesis**. (*A*) C57BL/6 wild-type and IL-37 transgenic mice were aged for 24 months, inoculated intravenously with BCR-ABL1^{+} *Arf-/-* murine B-ALL cells (mB-ALL) and (*B*) survival was monitored. Significance in *B* was determined using the log-rank test with ****p<0.0001. n=10 mice/group.

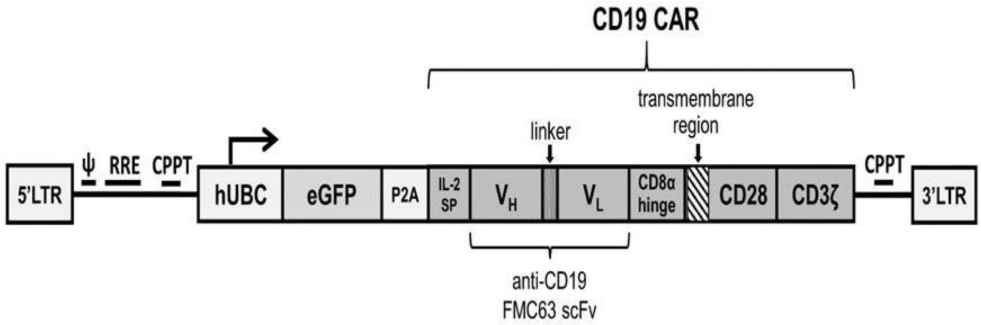

Figure S10. Schematic of the bicistronic construct encoding enhanced green fluorescent protein (eGFP) and the CD19-CAR. The transgene includes a 5’ long terminal repeat (LTR), human ubiquitin C promoter (hUBC), eGFP, a P2A sequence, the CD19-CAR and a 3’ LTR. Our second generation CD19-CAR consists of an interleukin-2 signal peptide (IL-2 SP), the anti-CD19 FMC63 single chain variable fragment (scFv), a CD8 alpha hinge region, the transmembrane and intracellular domains of CD28, and the CD3-ζ intracellular signaling domain.
