# Supplementary material for: Interleukin‐37 improves T‐cell‐mediated immunity and chimeric antigen receptor T‐cell therapy in aged backgrounds
Source: Aging Cell. 2021 Jan 22;20(2):e13309. doi: 10.1111/acel.13309 (PMC7884049; doi:10.1111/acel.13309)
Supplement: Supplementary file 2 — Supplementary Material [file ACEL-20-e13309-s002.pdf]

Supporting Supplemental Information

Experimental Procedures

Lentiviral Vector Production (CAR-T generation)

High-titer, recombinant, self-inactivating (SIN) HIV lentiviral vector was produced using a four-plasmid system. Briefly, the expression plasmid encoding the CD19-CAR construct as well as packaging plasmids containing the *gag*, *pol*, and *envelope* (VSV-G) genes were transiently transfected into HEK-293T cells by calcium phosphate transfection. Cells were cultured in Dulbecco's modified essential medium (DMEM, Thermo Fisher Scientific) supplemented with 10% FBS and 1% penicillin–streptomycin. Twenty-four hours after transfection, the cell culture medium was replaced with fresh medium. At 48, 72, and 96 hours the viral supernatant was collected, filtered through a 0.22 µm filter and stored at −80°C. After the final virus collection, the supernatant was pooled and concentrated overnight via centrifugation at 10,000g at 4°C. Pelleted virus was then resuspended in StemPro media (Thermo Fisher Scientific). Titering was performed on HEK-293T cells using quantitative polymerase chain reaction. Titer of the concentrated virus was found to be ~1×10^8 transducing units (TU)/mL.

Lentiviral Transduction of T-cells (CAR-T generation)

Human T-cells were isolated from cryopreserved peripheral blood mononuclear cells (PBMCs) purchased from AllCells (Alameda, CA) using EasySep Human T-cell Isolation Kit (Stem Cell Technologies, Cambridge, MA) or isolated from mice using magnetic-activated cell sorting (murine T-cells were $Ter119^-CD11c^-B220^-Mac\text{-}1^-Gr\text{-}1^-$ and $CD3^+CD4^+$ or $CD3^+CD8^+$). Isolated T-cells were resuspended in X-vivo 15 medium (Lonza, Basel, Switzerland) supplemented with 10% fetal bovine serum, 1% penicillin/streptomycin, 100 IU/mL of IL-2 and 5 ng/mL of IL-7 (Peprotech, Rocky Hill, NJ). T-cells were activated with CD3/CD28 Dynabeads (ThermoFisher Scientific, Waltham, MA) for 24 hours prior to transduction. Transduction of recombinant HIV lentiviral particles was carried out by incubating cells with the CD19-CAR encoding lentiviral vector in complete medium supplemented with 8 µg/ml polybrene (EMD Millipore, Billerica, MA) at a multiplicity of infection (MOI) of 25. Eighteen hours after transduction, media was replaced. The transduced cells were then cultured for at least 5 days prior to use in experiments, with media replacement occurring every 3 days.

TNF-α, IL-37, and qRT-PCR Analysis for TNF-α and PD-1 Target Genes

pNF-κB detection and PD-1 surface expression experiments. C57BL/6 mice were treated with control immunologlobin or rIL-37 for two weeks as previously described. After two weeks of treatment, spleens were harvested, and T-cell were isolated using MACs selection. $CD4^+$ or $CD8^+$ T-cells were stimulated (10^4-5×10^4 cells/well) in 96-well flat bottom plates (Millipore Sigma; cat. no. CLS3997) coated with αCD3 (10 µg/mL; BD Biosciences; cat. no. 553057) and αCD28 (2 µg/mL; BD Biosciences; cat. no. 553294) antibodies in RPMI 1640 media supplemented with Ctrl Ig (5 ng/mL) or IL-37 (5 ng/mL). The activation of NF-κB was determined after 4 hours of culture (phospho-NF-κB-PE; Cell Signaling Technology; product no. 5733S) and PD-1 surface expression on aged T-cells was determined on day 3 of culture.

qRT-PCR assays. Naïve T-cells were isolated from the mice treated as described above. T-cells were briefly cultured *in vitro* with Ctrl Ig (5 ng/mL) or IL-37 (5 ng/mL) for 4 hours and RNA was prepared per the manufacturer's instructions using the RNeasy Plus Micro Kit (Qiagen; Cat. No. 74034) followed by cDNA generation using the First Strand Synthesis Kit (Roche; Ref. No. 04 896 866 001). cDNA was diluted to 1:10 and 2µL of diluted cDNA was used to amplify genes of interest using the Thermo Fisher Scientific Gene Expression Assays per manufacturer's instructions:

*Actin (Actb; Cat. No. Mm02619580_g1). **Used as an internal control**.

*SHP2 (alias PTPN11; Ptpn11; Cat. No. Mm00448434_m1)

*GM130 (alias GOLGA2; Golga2; Cat. No. Mm00505620_m1)

*TMEM16F (alias ANO6; Ano6; Cat. No. Mm00614693_m1)

*PD1 (Pdcd1; Cat. No. Mm01285676_m1)

*IFN-γ (Ifng; Cat. No. Mm01168134_m1)

*IRF3 (Irf3; Cat. No. Mm00516784_m1)

*TBK1 (Tbk1; Cat. No. Mm00451150_m1)

All qPCR assays were performed using a LightCycler 480 (Roche).

qRT-PCR Analysis for T-cells Activation and Inhibitory Genes

C57BL/6 mice were treated with control immunologlobin or rIL-37 for two weeks as previously described. Splenic T-cells ($CD4^+$ and $CD8^+$) were isolated using MACs selection and RNA (1 µg) was prepared per the manufacturer's instructions using the Quick-RNA Miniprep Kit (Zymo Research, Cat. No. R1055). After RNA isolation, cDNA was generated using the High Capacity cDNA Reverse Transcription Kit (Applied Biosystems,™ Cat. No. 4368814) or using the Transcriptor First Strand cDNA Synthesis kit (Roche, Cat. No. 04 379 012 001). The cDNA was diluted to 1:10 and 5µL of diluted cDNA was used to amplify genes of interest using the PerfeCTa SYBR Green FastMix ROX Kit (Quanta BioSciences, Cat. No. 9503012) or the iTaq universal SYBR Green Supermix (Bio-Rad, Cat. No. 172-5122) per manufacturer's instructions. All qPCR assays were performed using a T100™ Thermal Cycler (Bio-Rad).

Flow Cytometric Analysis

Surface staining for immune cell distribution in the bone marrow and spleen. Single-cell suspensions were plated in 96-well round or flat-bottomed plates and washed in FACS buffer containing 3% FBS, 1× PBS, and 2 mM EDTA (vol/vol). After washing, cells were surface stained for 1 hour (covered on ice) to identify the hematopoietic cell population of interest (cells were stained in 50 µl Ab solution). Cells were washed once with 200 µl FACS buffer and resuspended in 400 µl FACS buffer for flow cytometric analysis. The following anti-mouse Abs were used:

Phycoerythrin-conjugated (PE-conjugated): anti-B220 (BD Pharmingen; catalog 553090), anti-CD43 (BD Pharmingen; catalog 553271), anti-CD3 (Biolegend; catalog 100206), anti-CD4 (Thermo Fisher Scientific; catalog 12-0041-82), anti-CD8 (Thermo Fisher Scientific; catalog 12-0081-82), anti-Ter119 (Thermo Fisher Scientific; catalog 12-5921-81), anti-CD48 (BD Pharmingen; catalog 557485), anti-Mac-1 (BD Pharmingen; catalog 557397), anti-Gr-1 (Biolegend; catalog 108408), anti-CD28 (BD Biosciences; catalog 561789), anti-CD8 (BD Biosciences; catalog 561095), anti-CD127 (Thermo Fisher Scientific; catalog 12-1271-82), anti-CD11b (BD Biosciences; catalog 101208), and anti-CD197 (CCR7) (Biolegend; catalog 120105)

PE-Cy7-conjugated: anti-Mac-1 (Thermo Fisher Scientific; catalog 25-0112-82), anti-CD49b (Thermo Fisher Scientific; catalog 25-5971-82), streptavidin (Thermo Fisher Scientific; catalog 25-4317-82), anti-Tim-3 (Biolegend; catalog 119716), and anti-PD-L1 (Invitrogen; catalog 25-5982-80)

Allophycocyanin-conjugated: anti-B220 (Thermo Fisher Scientific; catalog 17-0452-82), anti-Sca1 (Thermo Fisher Scientific; catalog 17-5981-82), anti-CD150 (Thermo Fisher Scientific; catalog 17-1502-80), and anti-CD8 (Biolegend; catalog 561093)

Pacific Blue-conjugated: streptavidin (e450) (Thermo Fisher Scientific; catalog 48-4317-82), anti-CD93 (Thermo Fisher Scientific; catalog 48-5892-80), anti-Flk2 (Flt3) (Cell Signaling Technology; catalog 28435S), anti-CD40 (Biolegend; catalog 124626), and anti-CD44 (Biolegend; catalog 103019)

FITC-conjugated: anti-c-KIT (BD Pharmingen; catalog 553354), biotin-linked anti-CD93 (Thermo Fisher Scientific; catalog 13-5892-82), anti-CD4 (BD Biosciences; catalog 553047), anti-KLRG1 (BD Biosciences; catalog 561619), anti-PD-L2 (eBioscience; catalog 11-9972-82), and anti-CD25 (IL-2R alpha) (Biolegend; catalog 101907)

PerCP-conjugated: anti-CD86 (Biolegend; catalog 105025) and anti-CD69 (Biolegend; catalog 104520)

APC-Cy7-conjugated: anti-CD4 (BD Biosciences; catalog 565650) and anti-CD62L (Biolegend; catalog 104427)

BV421-conjugated: anti-TIGIT (BD Biosciences; catalog 565270)

BV510-conjugated: anti-PD-1 (Biolegend; catalog 329931)

BV605-conjugated**:** anti-CD11c (BD Biosciences; catalog 563057)

Note: Naïve T-cells were isolated from mice treated for 2 weeks with control Ig or rIL-37. The naive T-cells used in these experiments were $CCR7^{hi}$, $CD44^{lo}$, $CD69^{neg}$, $CD62L^{hi}$, and IL-$7R^{hi}$ which was determined after MACs selection via flow cytometry. In naïve mice, aging is associated with the accumulation of virtual memory T-cells ($CCR7^{hi}$, $CD44^{hi}$, $CD69^{neg}$, $CD62L^{hi}$, IL-$7R^{hi}$, and IL-$15R^{hi}$), even in the absence of immunogenic exposure (Quinn et al., 2018). After MACs selection we found that greater than 95% of T-cells used in our assays were naïve T-cells and not aging-associated virtual memory cells. Furthermore, we included a CD25 stain to assess the immunophenotype of $CD4^+$ T-cells obtained after MACs purification. We found that 90-95% of the $CD4^+$ T-cells we isolated were $CD25^{lo}$ to negative, indicating that our studies were conducted primarily with naïve $CD4^+$ T-cells and not T-regulatory cells.

Hematopoietic Stem and Progenitor Cells (HSPCs)

*Hematopoietic Stem Cells (HSCs): Negative for lineage markers (Ter119, CD3, CD4, CD8, Gr-1, CD11b, B220), Sca-1^+, CD117 (c-kit)$^+$, $CD48^-$, $Flk2^-$, $CD150^+$

*B progenitor cells: Pro-B cells: $B220^{lo}$, $CD93^+$, $CD43^{hi}$; Pre-B cells: $B220^{lo}$, $CD93^+$, $CD43^{intermed/lo}$; Immature B cells: $B220^{hi}$, $CD93^-$, $CD43^-$

*Common Myeloid Progenitors (CMP): Negative for lineage markers (Ter119, CD3, CD4, CD8, CD11b, B220), Sca-1^{Neg}, CD117 (c-kit)$^+$, CD16/32^-, $CD34^+$

*Granulocyte Monocyte Progenitors (GMPs): Negative for lineage markers (Ter119, CD3, CD4, CD8, Gr-1, CD11b, B220), Sca-1^{Neg}, CD117 (c-kit)$^+$, CD16/32^+, $CD34^+$

Splenic Immune Cells

*T-helper cells ($CD4^+$ T-cells): $CD3^+CD4^+$ (negative for the myeloid markers CD11c, CD11b, Gr-1)

*Cytotoxic T-cells ($CD8^+$ T-cells): $CD3^+CD8^+$ (negative for the myeloid markers described above)

*B-cells: $CD19^+B220^{Int/high}$ (negative for CD3, CD4, CD8, CD49b, Gr-1)

*Macrophages: $CD11b^+$ (negative for CD3, CD4, CD8, B220, CD49b, Gr-1)

For all experiments, flow cytometric analysis was performed on a CyAn, Cytomics FC 500, or Cell Lab Quanta SC flow cytometer (all from Beckman Coulter).

ELISA for Cytokine Levels

Serum analysis. Serum was collected as previously described (Henry et al., 2015). Briefly, blood was isolated from mice using cardiac puncture (23-gauge needle with at 1 mL syringe) and transferred to a 1.5 mL Eppendorf tube. The tubes were left undisturbed at room temperature for 30 minutes. Samples were then centrifuged at 3000 rpm for 10 minutes, and serum (supernatant) was collected, and stored at -20°C until processing. ELISA analysis for TNF-α, IL-1β, and IL-6 was performed per the manufacturer's protocols (BD OptEIA; Mouse TNF-α ELISA Kit, Cat. No. 560478/ Mouse IL-1β ELISA Kit, Cat. No. 559603/ Mouse IL-6 ELISA Kit, Cat. No. 555240).

Interleukin-37 Data Mining of Human Samples

The R2 Database was utilized to determine the gene expression levels of IL-37 across age ranges. The database used was from Tompkins study, the total cellular RNA was extracted and hybridized onto HU133 Plus 2.0 GeneChip (Affymetrix). In this study, 37 healthy control samples were used to determine the expression levels of IL-37 in healthy donors. The GEO ID of the Tompkins study is gse36809 and PMID is 22110166.

IL-37 Gene Expression Assays of Human Monocytes

De-identified PBMCs were obtained from healthy donors of various ages (10-80 years of age) from the Children's Healthcare of Atlanta and Emory University's Children's Clinical and Translational Discovery Core. Monocytes were purified from peripheral blood mononuclear cells (PBMCs) using CD14 microbeads (Miltenyi Biotec, cat. no. 130-050-201). qPCR analysis was performed as described above using the IL-37-directed TaqMan® Gene Expression Assay (Assay ID Hs00367201_m1).

Table I. Sequences of qPCR primers

Genes	Sequences	Ref
IL-12r beta-1_F	CCCCAGCGCTTTAGCTTT	P.N.A.S., 108(36), 14885-14889, 2011
IL-12r beta-1_R	GCCAATGTATCCGAGACTGC	P.N.A.S., 108(36), 14885-14889, 2011
CD3_F	TCTCGGAAGTCGAGGACAGT	
CD3_R	CATCAGCAAGCCCAGAGTGA	
PD1_F	CCTGGTCATTCACTTGGGCT	
PD1_R	AAGGCGGCCTGTTTTTCAGT	
CD28_F	TGTCCAAAGCCTTTCGCTCT	
CD28_R	GGCATGCTCGGTACCAAAT	
LAT_F	ACCCTGACCTTGGAGACAGT	
LAT_R	GGAGCTTCCTCTCCATCCAC	
Perforin_F	ATTGACAACGCAGGTGTCCC	
Perforin_R	CCATACACCTGGCACGAACT	
STAT4_F	TTGGTGTGTTGATGCTGGCT	
STAT4_R	ACCCACTTGAGGCTTTCCTG	
GAPDH_F	CCAGCCTCGTCCCG TAGAC	Cell Communication and signaling, 13:14, 2015
GAPDH_R	CGCCCAATACGGCCAAA	Cell Communication and signaling, 13:14, 2015

REFERENCES

Henry, C. J., Casas-Selves, M., Kim, J., Zaberezhnyy, V., Aghili, L., Daniel, A. E., . . . DeGregori, J. (2015). Aging-associated inflammation promotes selection for adaptive oncogenic events in B cell progenitors. *J Clin Invest, 125*(12), 4666-4680. doi:10.1172/JCI83024

Quinn, K. M., Fox, A., Harland, K. L., Russ, B. E., Li, J., Nguyen, T. H. O., . . . La Gruta, N. L. (2018). Age-Related Decline in Primary CD8(+) T Cell Responses Is Associated with the Development of Senescence in Virtual Memory CD8(+) T Cells. *Cell Rep, 23*(12), 3512-3524. doi:10.1016/j.celrep.2018.05.057
